# HOXA3 functions as the on-off switch to regulate the development of hESC-derived third pharyngeal pouch endoderm through EPHB2-mediated Wnt pathway

**DOI:** 10.3389/fimmu.2023.1258074

**Published:** 2024-01-08

**Authors:** Yingjie Fu, Xueyan Zhang, Haibin Wu, Pingping Zhang, Shoupei Liu, Tingting Guo, Huanhuan Shan, Yan Liang, Honglin Chen, Jinghe Xie, Yuyou Duan

**Affiliations:** ^1^ Laboratory of Stem Cells and Translational Medicine, Institute for Clinical Medicine, the Second Affiliation Hospital, School of Medicine, South China University of Technology, Guangzhou, China; ^2^ Laboratory of Stem Cells and Translational Medicine, Institutes for Life Sciences, School of Medicine, South China University of Technology, Guangzhou, China; ^3^ Department of Laboratory Medicine, the Second Affiliation Hospital, School of Medicine, South China University of Technology, Guangzhou, China; ^4^ Medical Research Institute, Guangdong Provincial People’s Hospital (Guangdong Academy of Medical Sciences), Southern Medical University, Guangzhou, China; ^5^ School of Biomedical Sciences and Engineering, South China University of Technology, Guangzhou, China; ^6^ National Engineering Research Center for Tissue Restoration and Reconstruction, South China University of Technology, Guangzhou, China; ^7^ The Innovation Centre of Ministry of Education for Development and Diseases, the Second Affiliated Hospital, School of Medicine, South China University of Technology, Guangzhou, China

**Keywords:** human embryonic stem cell differentiation, third pharyngeal pouch, thymus, HOXA3, EPHB2, Wnt

## Abstract

**Objectives:**

Normal commitment of the endoderm of the third pharyngeal pouch (3PP) is essential for the development and differentiation of the thymus. The aim of this study was to investigate the role of transcription factor HOXA3 in the development and differentiation of 3PP endoderm (3PPE) from human embryonic stem cells (hESCs).

**Methods:**

The 3PPE was differentiated from hESC-derived definitive endoderm (DE) by mimicking developmental queues with Activin A, WNT3A, retinoic acid and BMP4. The function of 3PPE was assessed by further differentiating into functional thymic epithelial cells (TECs). The effect of HOXA3 inhibition on cells of 3PPE was subsequently investigated.

**Results:**

A highly efficient approach for differentiating 3PPE cells was developed and these cells expressed 3PPE related genes HOXA3, SIX1, PAX9 as well as EpCAM. 3PPE cells had a strong potential to develop into TECs which expressed both cortical TEC markers K8 and CD205, and medullary TEC markers K5 and AIRE, and also promoted the development and maturation of T cells. More importantly, transcription factor HOXA3 not only regulated the differentiation of 3PPE, but also had a crucial role for the proliferation and migration of 3PPE cells. Our further investigation revealed that HOXA3 controlled the commitment and function of 3PPE through the regulation of Wnt signaling pathway by activating EPHB2.

**Conclusion:**

Our results demonstrated that HOXA3 functioned as the on-off switch to regulate the development of hESC-derived 3PPE through EPHB2-mediated Wnt pathway, and our findings will provide new insights into studying the development of 3PP and thymic organ *in vitro* and *in vivo*.

## Introduction

The pharyngeal organs, namely the thyroid, thymus, parathyroid, and ultimobranchial bodies, are derived from the pharyngeal endoderm during the embryonic development. It is well known that Hox family genes control spatial identity along the anterior-posterior axis of the developing vertebrate embryo (1). Each pharyngeal arch is composed of cells from all three germ layers, including an external ectodermal layer, an internal endodermal layer, and a central mesenchymal core. HOXA3 was the first HOX gene to be mutated by gene targeting in mice and it was found to be required for the development of multiple endoderm and neural crest cell (NCC)-derived structures in the pharyngeal region (2). HOXA3 is expressed by the neural crest cells that originate from the rhombomeres, viz., (r)5, r6, and r7, and populate the third pharyngeal arch, and is also expressed in the third pharyngeal pouch (3PP) (1). HOXA3 is responsible for specifying organ identity within 3PP (1). It has been shown that HOXA3 null mutants lack 3PP derivatives like thymus and parathyroid glands and have alterations in the location and timing of key region markers within their 3PPs, including TBX1, BMP4, and FGF8 (2).

The thymus is a bilobed organ located directly above the heart in the chest, which is responsible for positive and negative selection of thymocytes, before mature T cells enter the peripheral blood circulation and exert immune functions (3). Thymic epithelial cells (TEC) are the main functional component of the thymus, all of which are derived from the endoderm of 3PP (4). During the embryogenesis, thymus parathyroid organogenesis is a highly dynamic process, and 3PP must undergo a complex series of morphogenetic events to detach from the pharynx and separate into different organ domains (5). Eventually the thymus is allowed to proceed inferiorly into the upper mediastinum towards the heart, while the parathyroid glands remain in the neck, associated with the thyroid gland. Whereas all of these events occur over a relatively short period of time.

HOXA3 is a key transcription factor gene in the primary regulation of 3PP endoderm (3PPE). HOXA3 was deleted in mice by homologous recombination (6), and together with HOXA1 (7), providing the first evidence implicating a role of HOX genes in pharyngeal development. It is well known that mouse embryos develop organ primordia from E10.5 to E13.5, including patterning and initial organogenesis. The spatiotemporal HOXA3 expression pattern in 3PP is at E10.5, HOXA3 expression is strong and its level in the endodermal epithelium is similar to that of surrounding neural crest-derived mesenchyme. At E11.0-E11.5, HOXA3 expression in the endoderm and pharyngeal pouches declines relative to that of surrounding NCCs. While the level of HOXA3 expression of NCCs began to decrease at E12.5. HOXA3 expression is then undetectable in either the endoderm or surrounding NCCs at E13.5 (2, 8). This dynamic change of HOXA3 in mice may affect changes in early and later organ morphogenetic events. It has been shown that Hoxa3 has cell type-specific roles during the third pharyngeal pouch development, and expressed in two of these cell types, the neural crest-derived mesenchyme, and the endodermal cells of the third pharyngeal pouch. Hoxa3 was primarily required in NCCs for the morphogenesis. In endoderm, Hoxa3 temporally regulated the initiation of the thymus program and was required in a cell-autonomous manner for the parathyroid differentiation (2). Genes that were expressed in each of these cell types were used as molecular markers to determine the earliest stage at which a defect in thymus development could be detected. Embryos were initially analyzed at E10.5 after neural crest migration was complete, but before overt thymus development was detectable (9, 10).

The 3PP appears at day 9 after mouse embryonic development (E9.0), and the size of 3PP begins to increase by E10.5. More dramatic changes occur in the following 24 hours at the cellular and molecular levels at E11.5. Differential proliferation within the primordium results in a larger prospective thymic (ventral) domain relative to the parathyroid (dorsal) domain at E11.5 (8). It has been well documented that HOXA3 plays an important role in mouse early embryonic development, including controlling processes such as cell migration, proliferation, differentiation and apoptosis, and that HOXA3 is in a dynamic change process in the formation of 3PP appearing to the thymus and parathyroid gland, however, the mechanism by which HOXA3 regulates cell migration and proliferation and differentiation remains unclear. Human embryonic stem cells (hESCs) provide an excellent *in vitro* tool for studying the induction of various cell types including TECs (11–20). However, the induction of 3PPE *in vitro* and the mechanism by which HOXA3 regulates the migration of 3PPE remains unknown. Here, we developed an approach to efficiently differentiate hESCs into 3PPE and probed the function of HOXA3 during the development and formation of 3PPE.

## Methods

### Human umbilical cord blood

Human umbilical cord blood was obtained from the donors with normal term delivery after signing informed consent. Cord blood sample collection was approved by the Ethics Committee of Guangzhou First People’s Hospital (Approval No K-2021-008-02) and complies with the Declaration of Helsinki.

### Human embryonic stem cell culture

hESCs, H9 line cells (WA09), were obtained from the WiCell Research Institute (Madison, WI, USA) with the Material Transfer Agreements (No.: 19-W0512, 24-W0162 and 24-W0163), and cultured and passaged on the feeder cells of mouse embryonic fibroblasts (MEF). The culture medium was DMEM-F12 (Gibco) basal medium supplemented with 20% knockout ™ Gibco), 4 ng/ml fibroblast growth factor bFGF (Peprotech), 100 mM L-glutamine (Gibco), 1% MEM non-essential amino acids solution (Thermo), 0.0007% β Mercaptoethanol. The medium was changed daily.

### Differentiation of hESCs into 3PPE

H9 cells were cultured on gelatin-coated plates with a feeder layer of MEF for 5 days, then the cells were subjected for endodermal differentiation. At the first day, differentiation medium was X-VIVO 20 medium (Lonza) supplemented with 100 ng/ml Activin A (Peprotech), 50 ng/ml WNT3A (Peprotech) or 3 μM CHIR99021(Selleck), 10 μM ROCK inhibitor Y-27632 (Selleck). The following day, the medium was changed to X-VIVO 20 (Lonza) medium containing 100 ng/ml Activin A, 0.5% B27 (Gibco). At day 4 after the differentiation, endodermal cells were dissociated with TrypLE (Gibco), and reseeded on Matrigel (Corning)-coated plates with DMEM-F12 medium (Gibco)or EGM2 medium (Lonza), supplemented with 0.5% B27, 1 μM RA (Sigma), 10 μM SB431542 (MCE), 20 ng/ml BMP4 (Peprotech), 2.5μM IWP2 (Selleck) or 2.5μM IWR1 (Selleck). The medium was changed daily, and the differentiation lasted until day 9 for the induction of 3PPE.

### Differentiation of thymic epithelial cells from 3PPE

For the differentiation of TECs, the differentiation from 3PPE cells were carried out from day 9 after the differentiation of 3PP cells employing DMEM-F12 medium supplemented with WNT3A (50 ng/mL), BMP4 (10ng/mL), FGF8 (50 ng/mL), FGF10 (5ng/mL) (all from Peprotech), Heparin sodium (10 ng/mL, GlpBio), SB431542 (10μM), SANT-1 (2.5 μmol/L) (selleck), 2-Phosphate-L-ascorbic acid trisodium salt (50 μg/mL, Sigma), B27 (0.5%, Gibco), RA (1μM, Sigma) for a total of 4 days, and the medium were changed once a day.

### qRT-PCR

Total RNAs were extracted by the Trizol (Takara) from hESCs that were differentiated at a specified time using a specified culture method. RNA concentration was measured using NanoDrop 2000 spectrophotometer (Thermo Fisher Scientific). According to the manufacturer’s instructions, 1000ng of RNAs were reversely transcribed into cDNAs using HiScript III RTSuperMix for qPCR (+gDNA wiper) kit (Vazyme). qRT-PCR was performed by using ChamQ Universal SYBR qPCR Master Mix (Vazyme) on QuantStudio (Applied Biosystems Life Technologies, Thermo Fisher Scientific). With GAPDH as internal parameter, the relative mRNA expression of samples was calculated by the 2^-ΔΔCt^ method. The primers used were listed in **Supplementary Table 3**.

### Immunofluorescence assays

The cells were inoculated and cultured in 12 well plates with cell slides which were placed before the inoculation for 1 days, then cells were fixed with 4% paraformaldehyde (PFA, biosharp) at room temperature for at least 15 minutes, and permeated with 0.5% Triton X-100 (Solarbio) for 20 minutes. Next the cells were blocked in goat serum (Boster) for 1 hour at room temperature, then incubate with the primary antibody at 4°C overnight. Next day, after rewarming at 37°C for 1 hour and washing, the cells were incubated with the secondary antibody at room temperature in dark for 2 hours. The cells were added with DAPI (Beyotime) to stain the nucleus and sealed with a fluorescent antiaging quenching agent (Beyotime). The images were collected by Zeiss 880 multiphoton laser scanning microscope or single photon confocal microscope (Nikon). All antibodies used were listed in **Supplementary Table 4**.

### Flow cytometry analysis

Cells were dissociated into single cells using TrypLE ™ Express Enzyme (1X) (Gibco), and the single cell suspension was collected and washed with PBS once for immunostaining. For the staining of the cell surface marker, the cells were directly stained and incubated at 4°C in dark for 30 minutes, and the dead cells were eliminated by DAPI or 7AAD (BD) staining. For the detection of nuclear transcription factors, FOXP3/Transcription factor staining buffer set (invitrogen) was used to fix and perforate cells. After cells were kept away from light at room temperature for 60 minutes, 1 x perm buffer was directly added to each tube to stop the reaction. The cells were then incubated at room temperature for 45 minutes with the primary antibody, afterwards PBS (Solarbio) was filled, the stained cells were centrifuged with 300g for 5 minutes, and with 8500g for 2 minutes after mixing. After cells were resuspended and incubated at room temperature for 45 minutes with the second-fluorescence antibody, then PBS (Solarbio) was filled, the stained cells were centrifuged with 300g for 5 minutes, and with 8500g for 2 minutes after mixing. Flow cytometry was performed with BD Celesta and BD Fortessa. Folwjo (V10) software was used for data analysis.

### Western blotting analysis

Cells were lysed by ultrasound in RIPA cell lysate buffer containing protease inhibitor and PMSF. The protein was harvested at 4°C by the centrifugation with 14000g for 15 minutes. According to the manufacturer’s protocol, BCA protein assay kit (Boster) was used for the determination of protein concentration. After protein quantification, protein denaturation was carried out and 20 µg proteins were added to 10% SDS-PAGE separation gel, and then transferred to PVDF membrane (Merck). The protein strips were sealed with 5% defatted milk powder (Solarbio) at room temperature for 1 hour, then incubated with the primary antibody, and incubated at 4°C overnight. Subsequently, the protein strips were washed by TBST (Solarbio) and incubated the second antibody at room temperature. One hour later, the expression of the target proteins was detected by the chemiluminescent ECL kit (Affinity).

### Knockdown assay by siRNA

Small interfering RNAs (siRNAs) against HOXA3 and EPHB2 were synthesized by Guangzhou Ige BIOTECHNOLOGY Co., Ltd, and the siRNA sequences for the negative control, HOXA3 and EPHB2 were listed in **Supplementary Table 3**. At day 7 after the differentiation, when the cell density reached 70% – 80%, the differentiated cells were transduced with HOXA3 siRNA or EPHB2 siRNA and negative control siRNA using Lipofectamine 3000 (Thermo Fisher Scientific), respectively. Six to eight hours after the transduction, the differentiation medium was replaced with opti-MEM medium (Thermo Fisher Scientific), and cells were harvested at days 9 after the differentiation. siRNAs were introduced into cells for transient transduction following the manufacturer’s instructions.

### Cell proliferation assay

The effects of HOXA3 and EPHB2 on cell proliferation were determined by a cell counting kit-8 assay (CCK-8; Dojindo). There were three groups including blank control group, NC group, siRNA-HOXA3 group or siRNA-EPHB2 group. Briefly, 5 × 10^3^ cells per well (in quadruplicate per group) were seeded into 96-well plates and cultured in differentiation medium. At 24, 48 and 72 hours after the culture, 10 μL of CCK-8 solution and 100 μL of fresh culture medium were added to each well and incubated at 37°C for additional 4 hours. The absorbance was observed at 450 nm by using a microplate reader (Amershan Iamger 600). The ratio of the survival/proliferation of cells was represented through the absorbance of the test wells minus the optical density of the blank wells.

### Wounding healing assay

For the scratch wound assay, 2 × 10^5^ cells per well (in triplicate per group) were plated into 12-well plates and incubated to reach confluence. The monolayer was scratched using a tip and washed with serum-free medium to remove detached cells. Cells were photographed at 0 and 24 hours post wounding. The closure area of wound was calculated by which migration area (%) = (A_0_ - An)/A_0_ × 100, where A_0_ represented the area of initial wound area, An represented the area of wound at the metering point.

### Migration assay

The migration assays were performed with the transwells, briefly 5 × 10^4^ cells per well were suspended in serum free medium and seeded into the upper chambers of 24-well transwell plates (Corning) with 8 μm pore filters, then the lower chambers were added with differentiation medium. At 48 and 72 hours after the culture, the cells attached on the upper surface of the filter membranes were removed and migrated cells of the lower surface were stained with 0.5% crystal violet for several minutes. The number of migrated cells was observed under an optical microscope (Nikon).

### Cell cycle analysis

Cell cycle distribution was determined by a cell cycle and apoptosis analysis kit (Meilunbio) according to manufacturer’s protocols. In brief, 3PPE cells were transduced with HOXA3 siRNA or EPHB2 siRNA and negative control siRNA for 48 hours, then these cells were fixed with 70% cold ethanol at 4°C overnight. The next day, the cells were washed with cold PBS and stained with the mixture of propidium iodide and RNase A. Afterwards, the cell cycle was detected by flow cytometry (BD Biosciences).

### RNA-seq

RNAs were extracted from cells of NC group or siRNA-HOXA3 group with triplicate for each group. 100ng of total RNAs was used to prepare sequencing libraries using the Ligation Mediated Sequencing (LM-Seq) protocol (21) and quantified with the Qubit 2.0 fluorometer (ThermoFisher). Final cDNA libraries were quantitated with the Agilent 2100 bioanalyzer, multiplexed and loaded at a final concentration of 2.5 nM, and sequenced as single reads on the Illumina NovaSeq 6000 (Illumina).

### Analyses for differentially gene expression and gene ontology enrichment

DESeq2 software was used to identify differentially expressed genes (DEGs). Empirical Bayesian methods are to estimate prior values of log-fold changes and deviations and to calculate posterior values of these statistics. DEGs were evaluated by meeting the two criteria, one included more than onefold change in expression and the other included pvalue <.05. For functional enrichment analysis, all DEGs were mapped to terms in the GO databases, and then significantly enriched GO terms were searched for among the DEGs using p <.05 as the threshold. GO term analysis was classified into three subgroups, namely biological process (BP), cellular component (CC) and molecular function (MF).

### Gene set enrichment analysis

GSEA (http://software.broadinstitute.org/gsea/index.jsp) was performed as previously described[26]. Analyzed gene sets (GOBP_NEGATIVE_REGULATION_OF_EPITHELIAL_CELL_MIGRATION, GOBP_POSITIVE_REGULATION_OF_EPITHELIAL_CELL_PROLIFERATION and WP_WNT_SIGNALING_PATHWAY_NETPATH) were downloaded from GSEA. To assess the enrichment, the absolute value of the GSEA metric (Pearson correlation) was considered for deregulated genes.

### Chromatin immunoprecipitation assay

The ChIP was achieved using ChIP Assay Kit (Chromatin Immunoprecipitation (ChIP) Assay Kit) (Beyotime). In brief, DNA and proteins were cross-linked with 37% formaldehyde at 37°C for 10 minutes. The 3PPE cells were added into glycine solution for 5 minutes at room temperature, then the cells were washed and collected using cold PBS solution supplemented with 1mM PMSF (Solarbio). The cell precipitates obtained after the centrifugation were resuspended by SDS lysis buffer containing 1mM PMSF, and fully lysed on ice for 10minutes. Next, cell lysates were dealt with pulses ultrasonication to break nuclear membrane and shear DNA into fragments with 200–1000 bp fragments. The final lysates were incubated with 1 μg HOXA3 antibody (Santa Cruz) or 1 μg mouse immunoglobulin G (IgG) antibody (Beyotime) at 4°C overnight. All told, 10 μg chromatin was used as input control and mouse anti-IgG was used as negative control. The precipitated DNAs were subjected to PCR analysis using a primer pair specific for EPHB2. The amplified fragments were analyzed by 1.5% (w/v) agarose gel analysis and verified by qRT-PCR.

### Analysis of single cell sequencing

Raw data for single-cell sequencing provided by Margaret E. Magaletta et al (22). The scRNA-seq short reads and count matrices data were used in this study from the Gene Expression Omnibus (GEO) database under accession codes “GSE182135”. The atlas had a total of 57717 cells and ten samples (E9.5:2; E10.5:3; E11.5:3; E12.5:2). A typical Seurat workflow was followed, using gene selection (with FindVariableGenes), scaling (with ScaleData), and principal components analysis (PCA with RunPCA). For this initial analysis, 50 principal components (PCs) were retained for downstream steps, and the Louvain graph clustering (via FindClusters) was used with a resolution of 2 graph-based clustering with the Louvain algorithm with FindClusters, and UMAP visualization with RunUMAP. Out of the resulting 47 clusters, we removed cluster 12, 23, 27, 29, 31, 32, 36, 38, 39, 41, 42 and 44 due to low Pax9 and Epcam (associated with neuronal signature). The 49,087 cells passing quality control were analyzed using Seurat to produce overview figures. 60 principal components (PCs) were retained for downstream steps, and the Louvain graph clustering (via FindClusters) was used with a resolution of 0.5. The code and documentation are available at the GitHub repositories [https://github.com/fyj3600/HOXA3/tree/fyj3600-patch-1].

### Statistical analysis

All statistical analyses were performed using the SPSS 13.0 package (SPSS International, Chicago, IL, USA). Continuous variables were presented as mean ± SD and analyzed using the Student’s t-test or ANOVA (analysis of variance).

## Results

### Hoxa3 was dynamically altered at the pharyngeal pouch stage in mouse embryos

**Figure 1:**
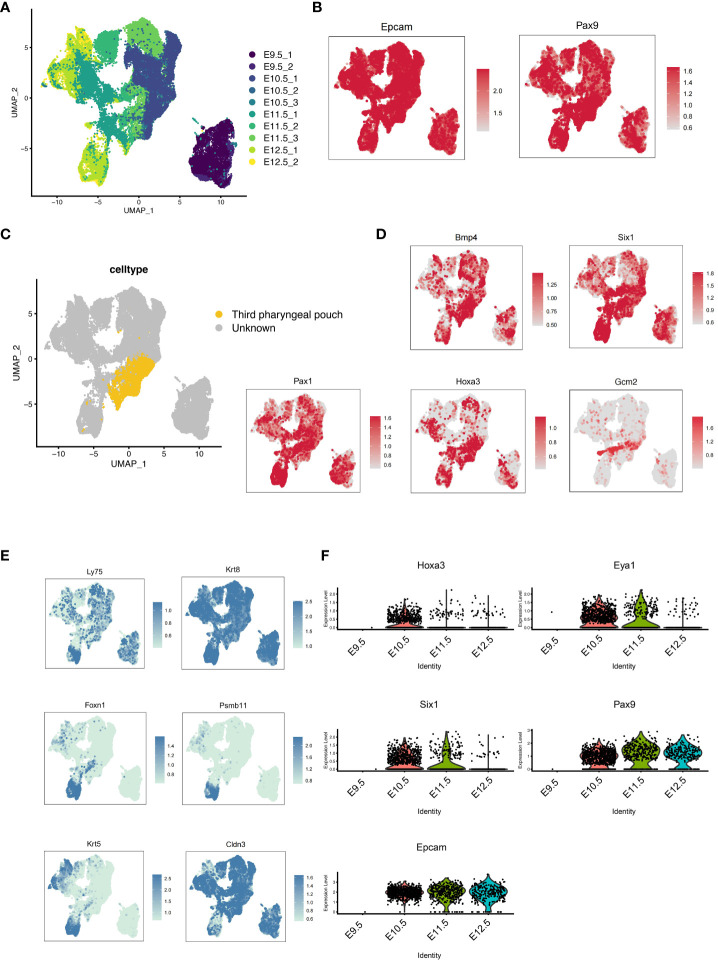
Development of the pharyngeal pouch in a mouse embryo by single-cell transcriptome sequencing. **(A)** Pax9^+^Epcam^+^ cells in mouse embryos at E9.5, E10.5, E11.5, and E12.5 were analyzed by multisample integration. UMAP visualization of Pax9^+^Epcam^+^ cells colored by age group. UMAP visualization of pharyngeal endoderm transcriptomic time-course dataset (n=53,693 cells) colored by embryonic day. **(B)** The UMAP maps showed the expression levels of specific pharyngeal endoderm markers Pax9 and Epcam. **(C)** The UMAP showed the third pharyngeal pouch cells within the Pax9^+^Epcam^+^ population. The yellow dots represented the third pharyngeal pouch cells. **(D)** The UMAP maps showed known markers of 3PPE. **(E)** The UMAP maps showed known markers of thymic epithelial cells. **(F)** Violin plots showed the dynamics of key genes of cell cluster in 3PPE.

To investigate the molecular processes underlying the development of pharyngeal endoderm, we analyzed a single-cell transcriptomic catalog (22) of mouse pharyngeal endoderm between embryonic days E9.5 and E12.5 which covers the transition from pharyngeal endoderm to pharyngeal organ primordia (23). To specifically isolate the pharyngeal endoderm, Epcam/Pax9^VENUS^ cells were sorted as previously described (22). After the initial data processing and quality control of the single-cell RNA (scRNA) profiles (see “Methods”), the resulting dataset yielded 49,087 single-cell transcriptomes (9,626 from E9.5, 11,230 from E10.5, 17,155 from E11.5 and 11,076 from E12.5). First, the single cell transcriptome data of mouse pharyngeal endoderm between embryonic days E9.5 and E12.5 were dimensional reduction and unsupervised clustering (**Figure 1A**; **Supplementary Figure 1A**). We performed UMAP display of the pharyngeal endoderm specific genes Pax9 and Epcam, and the results showed that almost all cells expressed Pax9 and Epcam (**Figure 1B**). Importantly, known markers were used to identify 3PP (24), including Bmp4, Six1, Pax1, Hoxa3 and Gcm2 genes. We identified 27 distinct clusters (**Supplementary Figure 1A**), then we analyzed the expression levels of related markers of 3PPE in each cluster using violin plots (**Supplementary Figure 1B**). 3PPE cluster was identified by manual inspection of differentially expressed genes for Hoxa3, Bmp4, Foxn1 and Gcm2. Finally, 8, 13 and 14 clusters were defined as 3PPE cluster (**Figures 1C, D**; **Supplementary Figure 1B**). The results indicated that Bmp4 and Six1 were not specific to 3PP, Pax1 was also not restricted to 3PP, and Gcm2 was expressed in the parathyroid domain of the 3PP or parathyroid, although all these markers were expressed in 3PP (**Figure 1D**). In parallelly, we also characterized key genes of the thymic epithelium, and the results showed that Cldn3, Ly75, Krt8 were more widely distributed in the pharyngeal pouches, but Foxn1, Psmb11 and Krt5 were specifically highly expressed at day E12.5 in mouse embryos (**Figure 1E**). We subsequently performed further analysis of these genes for Hoxa3, Eya1, Six1, Epcam as well as Pax9. It was worth noting that these genes presented a dynamic change pattern (**Figure 1F**). In particular, the expression of Hoxa3 in 3PPE cell cluster was increased rapidly to the highest level at day E10.5 in mouse embryos, followed by a rapid decrease (**Figure 1F**). At the same time, the dynamic changes in the expression levels of Pax9 and Six1 as well as Eya1 were also similar to those of Hoxa3 (**Figure 1F**). These results indicated that the key transcription factors of the third pharyngeal pouch showed a dynamic change trend of initial increase and later decrease in a very short period of time during mouse embryonic development.

### Differentiation of hESCs into 3PPE

**Figure 2:**
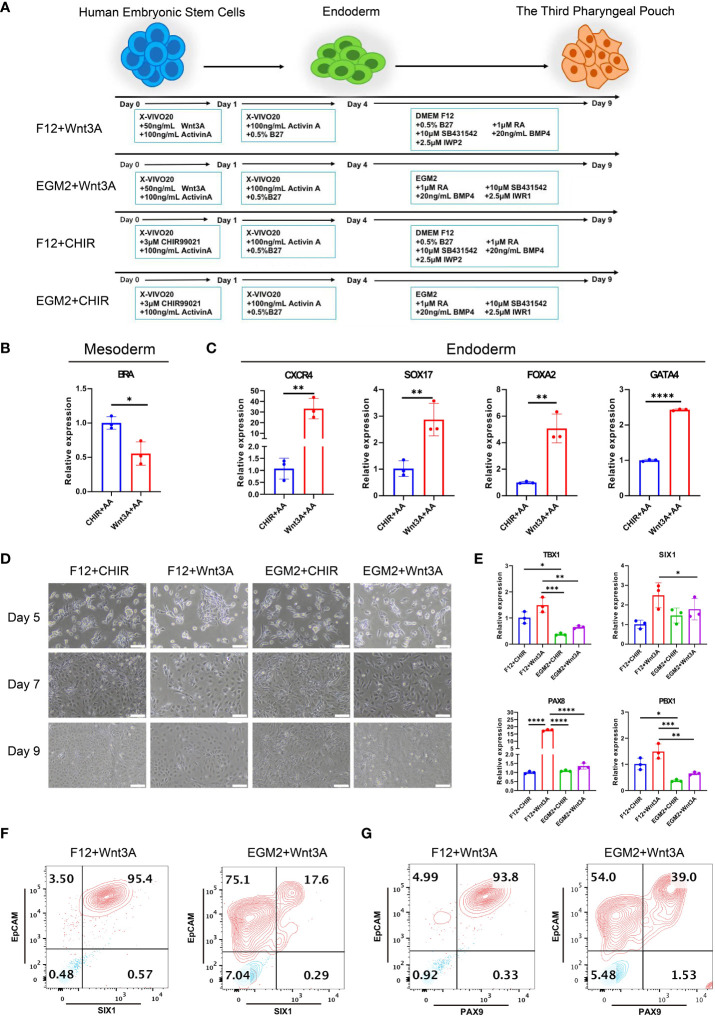
Differentiation of 3PPE from hESCs. **(A)** Schematic representation of different approaches for the differentiation of hESCs into 3PPE. **(B)** The relative expression levels of mesoderm marker Brachyury (BRA) were determined by qRT-PCR at day 4 after the differentiation. qRT-PCR data were shown normalised to the CHIR+AA condition. **(C)** The relative expression levels of endoderm markers were determined by qRT-PCR at day 4 after the differentiation. qRT-PCR data were shown normalised to the CHIR+AA condition. **(D)** The dynamic changes of cell morphologies during the differentiation at days 5, 7 and 9. Scale bar = 100 µm. **(E)** The relative expression levels of markers of 3PPE were determined by qRT-PCR at day 9 after the differentiation. qRT-PCR data were shown normalised to the F12+Chir condition. **(F)** The proportions of SIX1 and EpCAM double positive cells were measured by flow cytometry at days 9 after the differentiation. **(G)** The proportions of PAX9 and EpCAM double positive cells were measured by flow cytometry at days 9 after the differentiation. Values were presented in mean ± SD. *p < 0.05, **p < 0.01, ***p < 0.001, ****p < 0.0001. n = 3 independent biological repeats.

To investigate the role of HOXA3 during the development of 3PPE, we established an approach to differentiate 3PPE from hESCs. We developed four different methods to differentiate hESCs and thus explored the most efficient one for the differentiation for 3PPE (**Figure 2A**). During the differentiation of definitive endoderm (DE), we used the classical DE differentiation approach with different combinations of Activin A (19), WNT3A and Activin A (11), or CHIR99021 and Activin A (25). At day 4 after the differentiation, the cells were examined by RT-qPCR, and the results showed that mesodermal marker Brachyury (BRA) was significantly downregulated (**Figure 2B**), and DE markers CXCR4, SOX17, FOXA2, and GATA4, were significantly upregulated in cells treated with the combination with WNT3A and Activin A when compared to those with combination with CHIR99021 and Activin A (**Figure 2C**). Thus, these results indicated that the combination with WNT3A and Activin A was more oriented toward definitive endodermal commitment.

Immediately afterwards, we continued the induction of endodermal cells toward 3PPE using EGM2 medium or DMEM-F12 medium supplemented with 0.5% B27, 1 μM RA, 10 μM TGF-β Inhibitor SB431542, 20ng/ml BMP4, 2.5 μM IWP2 or 2.5 μM IWR1. During the induction, the morphology of cells induced by the combination of DMEM-F12 and WNT3A was more homogeneous (**Figure 2D**), while cells with multiple morphologies were produced using EGM2 medium. Whereupon the cells were assayed at day 9 after the differentiation by qRT-PCR, and results showed the highest expression levels of key transcription factors TBX1, SIX1, PAX8 and PBX1 were obtained in 3PPE under the condition with DMEM-F12 and WNT3A (**Figure 2E**). Meanwhile, flow cytometry analysis also exhibited that the culture condition with the combination of DMEM-F12 and WNT3A produced 95% of SIX1^+^EpCAM^+^ cells at day 9 after the differentiation, while only 17% of the same cells were produced with the combination of EGM2 and WNT3A (**Figure 2F**; **Supplementary Figure 2A**). Additionally, the similar proportion of PAX9^+^EpCAM^+^ cells produced by the combination of DMEM-F12 and WNT3A was also much higher than that of the combination of EGM2 and WNT3A (**Figure 2G**; **Supplementary Figure 2B**). Therefore, an efficient approach for directed differentiation into 3PPE from hESCs was eventually determined.

### Characterization of hESC-derived endoderm and highly efficient generation of 3PPE from hESCs

**Figure 3:**
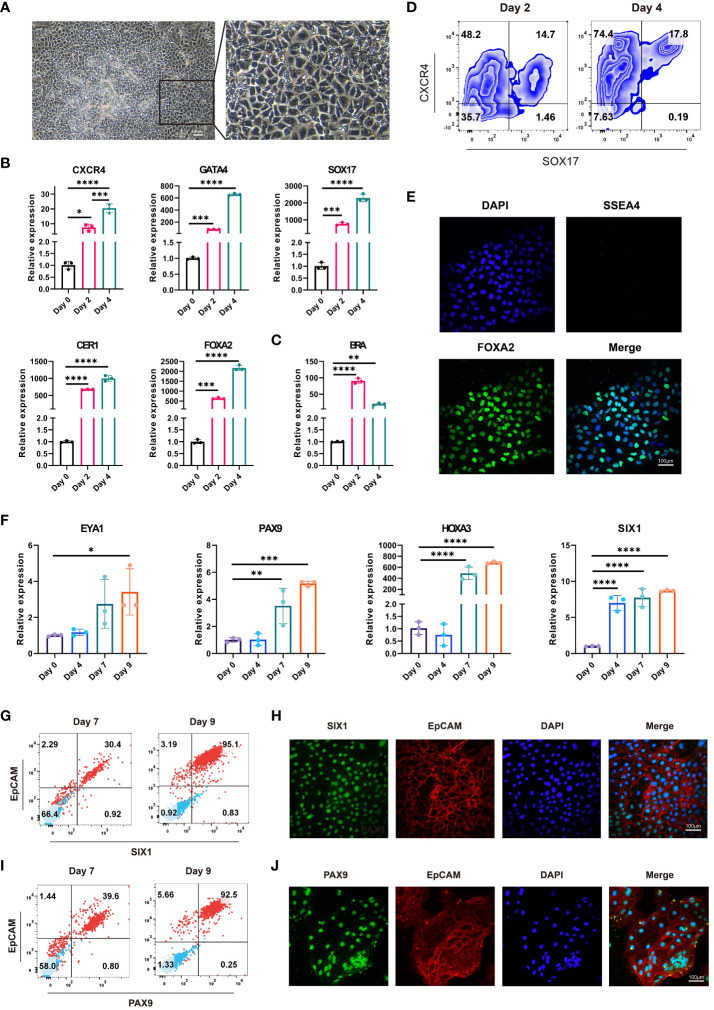
Identification and characterization of endodermal cells and 3PPE derived from hESCs. **(A)** The cell morphologies during the differentiation of hESCs at day 4. Scale bar = 100 µm. **(B)** The relative expression levels of endoderm markers were determined by qRT-PCR from day 0 to day 4 during the differentiation. **(C)** The relative expression level of mesoderm marker gene was determined by qRT-PCR from day 0 to day 4 during the differentiation. **(D)** The proportions of CXCR4 and SOX17 double positive cells were measured by flow cytometry at days 2 and 4 after the differentiation. **(E)** Co-immunostaining images of endoderm marker FOXA2 (Green) and pluripotent gene SSEA4 (Red), and DAPI was used to stain the nucleus (Blue). Scale bar = 100 µm. **(F)** The relative expression levels of marker genes of 3PPE were detected by qRT-PCR from day 0 to day 9 during the differentiation. **(G)** The proportions of SIX1 and EpCAM double positive cells were measured by flow cytometry at days 7 and 9 after the differentiation. **(H)** Double immunostaining images of markers EpCAM (Red) and SIX1 (Green) for 3PPE. DAPI was used to stain the nucleus (Blue). Scale bar = 100 µm. **(I)** The double positive proportion of cells for PAX9 and EpCAM were measured by flow cytometry at days 7 and 9 after the differentiation. **(J)** Double immunostaining images of marker EpCAM (Red) and PAX9 (Green) for 3PPE, and DAPI was used to stain the nucleus (Blue). Values are presented in mean ± SD. *p < 0.05, **p < 0.01, ***p < 0.001, ****p < 0.0001. All qRT-PCR data were shown normalised to the day 0 condition. n = 3 independent biological repeats.

The successful differentiation of the endoderm lays a tamped-down foundation for the formation of 3PPE. Under the microscope, we observed the uniform morphology of endodermal cells, which had clear cell borders, gray brown cytoplasm, a uniform shape in size and were arranged in a single layer (**Figure 3A**). To further characterize the endodermal cells, we collected cells at days 2 and 4 for analysis, and found that positive cell proportion for the pluripotent OCT4 gene was dramatically decreased (**Supplementary Figure 3A**), and the expression levels of endodermal genes CXCR4, GATA4, SOX17, CER1, FOXA2 all were gradually increased over time after the differentiation (**Figure 3B**), whereas the expression level of mesodermal gene BRA was first up-regulated then down-regulated (**Figure 3C**). In addition, the proportions of endodermal SOX17^+^ cells and CXCR4^+^ cells were increased from 16.1% to 18.0% (**Supplementary Figure 3B**), and 62.2% to 92.0% (**Supplementary Figure 3C**) respectively, and the proportion of CXCR4^+^SOX17^+^ double positive cells were increased from 14.7% to 17.8% (**Figure 3D**; **Supplementary Figure 3D**). Moreover, co-immunofluorescent staining analysis further verified that the endodermal cells expressed the endodermal marker FOXA2, but did not express the pluripotent gene SSEA4 at day 4 after the differentiation (**Figure 3E**).

Next, the endodermal cells were further induced toward 3PPE cells, and qRT-PCR was performed to determine the gene expressions of cells at days 0, 4, 7, and 9 during the induction. As expected, the expression levels of 3PPE marker genes EYA1, PAX9, HOXA3, and SIX1 were all highly expressed over time during the entire differentiation from day 0 to day 9 (**Figure 3F**). Moreover, the positive cell proportions of SIX1, a key marker of 3PPE, and EpCAM, a surface marker of epithelial cells, were determined by flow cytometry, and the results showed that SIX1^+^ cell proportions were increased from 31.4% to 96.6% at day 9 (**Supplementary Figure 4A**), and the proportions of SIX1^+^EpCAM^+^ double positive cells were increased from 30.4% to 95.1% at day 9 (**Figure 3G**; **Supplementary Figure 4C**), indicating that pure population of 3PPE were generated. Meanwhile, these results were also verified by co-immunofluorescent staining for SIX1 and EpCAM (**Figure 3H**). Next, we also found that the proportions of cells positive for PAX9, another key marker of 3PPE, was increased from 40.0% to 95.3% (**Supplementary Figure 4B**), while the proportions of double positive cells for PAX9 and EpCAM was increased from 39.6 to 92.5% at day 9 (**Figure 3I**; **Supplementary Figure 4D**), and these results were also verified by co-immunofluorescent staining for PAX9 and EpCAM (**Figure 3J**), highly consistent with those of SIX1 and EpCAM. Overall, we established a highly efficient approach to differentiate hESCs into 3PPE.

### Differentiation of 3PPE cells into TECs

**Figure 4:**
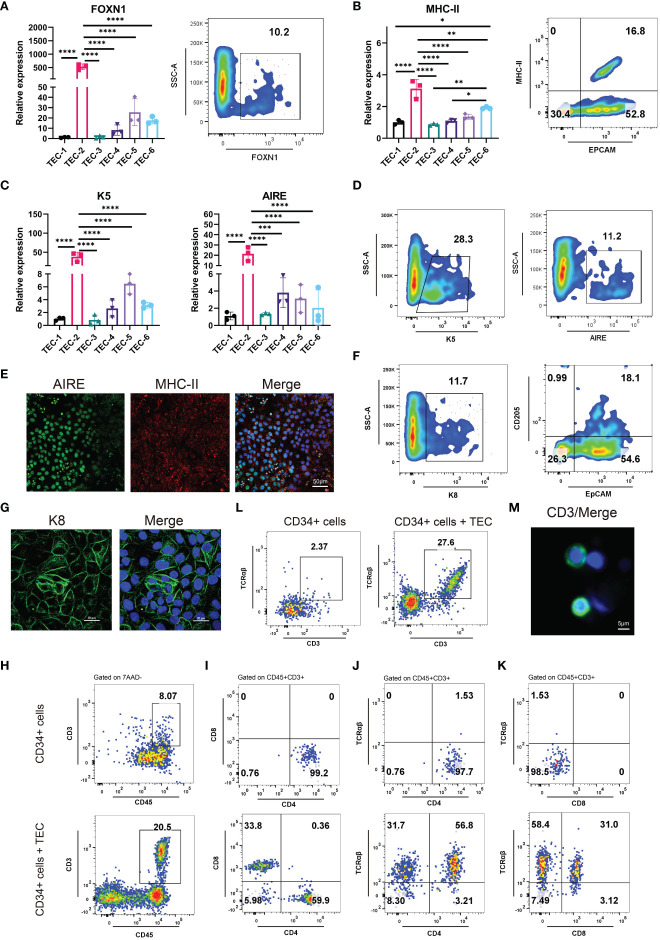
Differentiation of 3PPE into TECs with the capacity to promote T cell production. **(A)** The expressions of transcriptional regulator FOXN1 of TECs was detected by qRT-PCR (Left panel) under six different TEC culture conditions and by flow cytometry (Right panel) under TEC-2 culture condition respectively at day 13. **(B)** The expressions of mature TEC marker HLA-DRA (MHC class II molecule) and EpCAM were detected by qRT-PCR (Left panel) under six different TEC culture conditions and by flow cytometry (Right panel) under TEC-2 culture condition respectively at day 13. **(C)** The expressions of medullary TEC marker genes K5 and Aire were determined by qRT-PCR under six different TEC culture conditions at day 13. **(D)** The positive proportions of cells for medullary TEC markers K5 and Aire were measured by flow cytometry under TEC-2 culture condition at day 13. **(E)** Double immunostaining images of medullary TEC markers for MHC-II (Red) and Aire (Green), and DAPI was used to stain the nucleus (Blue). Scale bar = 50 µm. **(F)** The proportions of K8^+^ cells, CD205^+^ EpCAM^+^ cells were measured by flow cytometry under TEC-2 culture condition at day 13. **(G)** Immunostaining images of the cortical TEC marker K8 (Green), and DAPI was used to stain the nucleus (Blue). Scale bar = 20 µm. **(H)** Frequencies of mature CD45^+^CD3^+^ T cells were measured by flow cytometry in the differentiated cells of human HSPCs (Upper panel) and human HSPCs co-cultured with hESC-derived TECs (Bottom panel) (gated on 7AAD- cells). **(I)** Representative differentiation pattern of mature CD4^+^SP T cells and CD8^+^SP T cells were determined in CD45^+^CD3^+^ T cells by flow cytometry after the differentiation of HSPCs (Upper panel) and the co-culture of hESC-derived TECs with HSPCs (Bottom panel). Total live cells were presented in the 7AAD-gate. Subsequent parent gates were presented above each panel. **(J)** Representative differentiation pattern of mature TCRαβ^+^CD4^+^ T cells were determined in CD45^+^CD3^+^ T cells by flow cytometry after the differentiation of HSPCs (Upper panel) and the co-culture of hESC-derived TECs with HSPCs (Bottom panel). Total live cells were presented in the 7AAD-gate. Subsequent parent gates were presented above each panel. **(K)** Representative differentiation pattern of mature TCRαβ^+^CD8^+^ T cells were determined in CD45^+^CD3^+^ T cells by flow cytometry after the differentiation of HSPCs (Upper panel) and the co-culture of hESC-derived TECs with HSPCs (Bottom panel). Total live cells were presented in the 7AAD-gate. Subsequent parent gates were presented above each panel. **(L)** Frequencies of mature CD3^+^TCRαβ^+^ T cells were measured by flow cytometry in the differentiation of HSPCs (Upper panel) and the co-culture of hESC-derived TECs with HSPCs (Bottom panel) (gated on 7AAD^-^CD45^+^ cells). **(M)** Immunostaining images of T cell marker CD3 (Green), and DAPI was used to stain the nucleus (Blue). Scale bar = 5 µm. qRT-PCR data were shown normalised to the TEC-1 condition. n = 3 independent biological repeats. Values were presented in mean ± SD. *p < 0.05, **p < 0.01, ***p < 0.001, ****p < 0.0001.

We developed six differentiation culture conditions to induce TECs (**Supplementary Table 1**), and screened out the optimal culture one for the efficient differentiation towards TECs. Briefly, the differentiation medium for TECs was employed directly to 3PPE cells for a 4-day period at day 9 after the differentiation. At day 13 after the differentiation to TECs, the cells were collected for the analysis by RT-qPCR, and we found that the relative expression of FOXN1, a key transcription factor of TECs, was extremely higher under TEC-2 culture condition with over 10% of FOXN1^+^ cells in the differentiated cells when compared to those in other five culture conditions (**Figure 4A**; **Supplementary Figure 5A**). MHC-II, a specific marker of mature TECs, was also up-regulated with the highest expression levels in differentiated cells under TEC-2 culture condition (**Figure 4B**; **Supplementary Figure 5B**). Importantly, the proportion of MHC-II^+^EpCAM^+^ mature TECs could be reached to 16.8% (**Figure 4B**). TECs are usually divided into medullary TECs and cortical TECs. The former plays a role in the positive selection of T cells, while the latter makes negative selection of T cells and retains T cells that have no immunogenicity to host cells. We further found that the specific marker K5 of medullary TECs and the autoimmune regulator AIRE had the highest expression levels respectively in differentiated cells under TEC-2 culture condition (**Figure 4C**). Furthermore, we determined over 28% cells positive cells for K5 and 11% for AIRE by flow cytometry (**Figure 4D**; **Supplementary Figure 5C**), and a large number of cells co-expressed MHC-II and AIRE using immunofluorescent staining (**Figure 4E**; **Supplementary Figures 5F, G**), demonstrating that 3PPE cells had the ability to differentiate into medullary TECs. In addition, three major cortical TEC markers were also determined. Using flow cytometry, we found that the differentiated cells had 25% proportion of K8^+^ cells, and 18% of CD205^+^ and EpCAM^+^ cells, respectively (**Figure 4F**; **Supplementary Figures 5A, E**), and the expression of K8 was further verified by immunofluorescent staining (**Figure 4G**). To further demonstrate the role in promoting T cell development, we co-cultured hESC-derived TECs with human hematopoietic stem/progenitor CD34^+^ cells (HSPC), and the cells were analyzed by flow cytometry one week after the co-culture. Surprisingly, over 20% of double positive CD45^+^CD3^+^ T cells were generated (**Figure 4H**; **Supplementary Figure 5H**), almost 60% for CD4 single positive (SP) T cells, and over 30% for CD8 single positive T cells were included in CD45^+^CD3^+^ T cells (**Figure 4I**, bottom panel). On the contrary, without the microenvironment support provided by TECs, only 8% of CD45^+^CD3^+^cells were differentiated from CD34^+^cells (**Figure 4H**), and all of them were induced to CD4^+^SP cells, and none of CD8^+^SP cells was generated (**Figure 4I**, upper panel). Moreover, we further found that the proportions for TCRαβ^+^CD4^+^ double positive (DP) T cells was 56.8% (**Figure 4J**, bottom panel), and for TCRαβ^+^CD8^+^DP T cells was 31.0% in CD45^+^CD3^+^ T cells in co-culture condition (**Figure 4K**, bottom panel). However, without the microenvironment support provided by TECs, either CD4^+^SP cells or CD8^+^SP cells did not express TCRαβ (**Figures 4J, K**, upper panel). Surprisingly, we found that mature CD3^+^TCRαβ^+^ DP T cells accounted for 27.6% in the total CD45+ cells (**Figure 4L**; **Supplementary Figure 5H**), and CD3 positive cells were further verified using immunofluorescent staining (**Figure 4M**). Consistently, without the microenvironment support provided by TECs, only 2% CD3^+^TCRαβ^+^ were produced (**Figure 4L**). Collectively, our results not only demonstrated that 3PPE could be sufficient for further differentiation into functional TECs, also laid the fundamental basis for our further mechanism studies.

### HOXA3 regulated the differentiation, migration, proliferation and cell cycle of 3PPE

**Figure 5:**
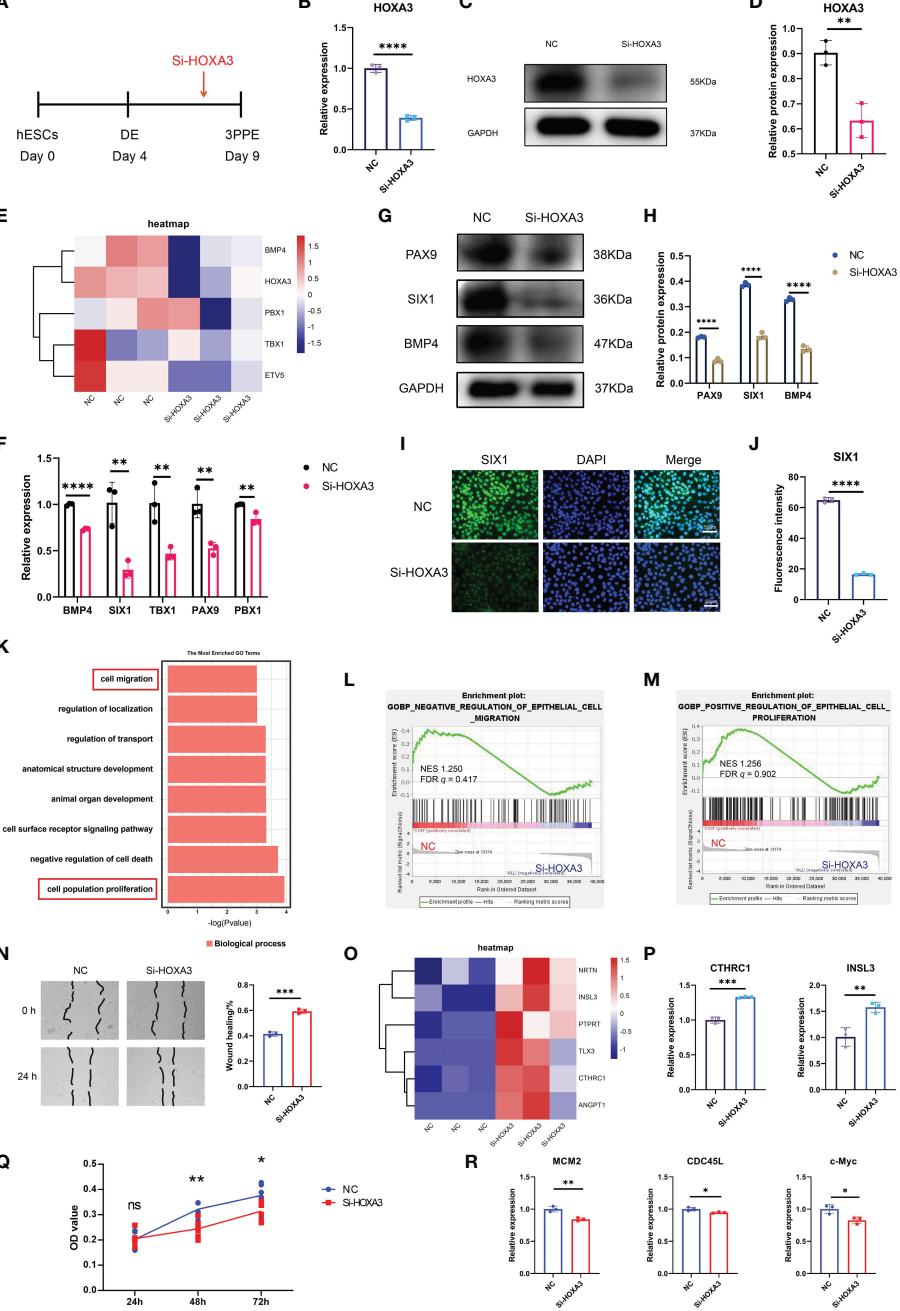
HOXA3 regulated the differentiation, migration and proliferation of 3PPE. **(A)** Schematic representation of siRNA transduction during the differentiation of 3PPE from hESCs. **(B)** The expression of HOXA3 gene was assessed by qRT-PCR after the treatment with HOXA3 siRNA. **(C, D)** Western blot was used to evaluate the protein expression of HOXA3 in cells treated with HOXA3 siRNA **(C)**, and protein levels were normalized to those of housekeeping gene GAPDH **(D)**. **(E)** Heatmap showed the transcriptional changes of key genes in 3PPE after the treatment with HOXA3 siRNA. **(F)** The expression changes of BMP4, SIX1, TBX1, PAX9, PBX1 were determined by qRT-PCR in cells treated with HOXA3 siRNA. **(G, H)** Western blot was used to evaluate the protein expressions of PAX9, SIX1 and BMP4 in cells treated with HOXA3 siRNA **(C)**, and protein levels were normalized to those of housekeeping gene GAPDH **(D)**. **(I)** Representative confocal images of immunostainings of SIX1 in cells treated with HOXA3 siRNA, and DAPI was used to stain the nucleus (blue). Scale bar = 50 µm. **(J)** The immunofluorescent quantifications of staining for SIX1 protein **(J)** in **(I)**. **(K)** The differentially expressed genes under HOXA3 inhibition showed biological process by GO function enrichment analysis after the treatment with HOXA3 siRNA. **(L)** GSEA analysis showed that the genes negatively regulating epithelial cell migration were down-regulated after HOXA3 inhibition with a normalized enrichment score of 1.250 (FDR *q* value of 0.417). **(M)** GSEA analysis showed that the genes regulating epithelial cell proliferation were down-regulated after HOXA3 inhibition with a normalized enrichment score of 1.256 (FDR *q* value of 0.902). **(N)** Representative images (Left panel) and quantitation (Right panel) of wound-healing assay for the measurement of cell migration 24 hours after the treatment with HOXA3 siRNA. **(O)** Heatmap exhibited transcriptional changes of genes associated with cell migration in 3PPE after the treatment with HOXA3 siRNA. **(P)** The expression changes of CTHRC1 and INSL3 were assessed by qRT-PCR in cells treated with HOXA3 siRNA. **(Q)** Cell proliferation assay was determined by CKK8 at 24, 48 and 72 hours after the treatment of cells with HOXA3 siRNA. **(R)** The expression changes of proliferation related genes MCM2, CDC45L and c-Myc was assessed by qRT-PCR 48 hours after the treatment of cells with HOXA3 siRNA. Values were presented in mean ± SD. *p < 0.05, **p < 0.01, ***p < 0.001, ****p < 0.0001. qRT-PCR data were shown normalised to the NC condition. n = 3 independent biological repeats.

Given that HOXA3 expression was associated with the differentiation and formation of 3PPE, we wondered whether HOXA3 inhibition would affect the development of 3PPE. To this end, we used siRNA against HOXA3 during the differentiation of hESCs into 3PPE at day 7 (**Figure 5A**), and we found that HOXA3 was significantly reduced both at mRNA expression level (**Figure 5B**) and protein expression level (**Figures 5C, D**). This result was also verified by immunofluorescence analysis in which HOXA3 protein expression was relatively weak (**Supplementary Figures 6A, B**). Next, total RNAs of the cells was subjected to RNA sequencing, and the Heatmap showed that the expression levels of key transcription factors of 3PPE were all downregulated when HOXA3 was repressed (**Figure 5E**). In addition, we also further validated the results by qRT-PCR, and found that the repression of HOXA3 led to the downregulation of BMP4, SIX1, TBX1, PAX9, PBX1 (**Figure 5F**), and that protein levels also declined in synchrony (**Figures 5G, H**). Immunofluorescent staining was performed to further verify these findings, and we observed diminished immunofluorescent intensity of transcription factors SIX1 (**Figures 5I, J**) and PAX9 (**Supplementary Figures 6C, D**) upon HOXA3 inhibition. Thus, these lines of evidence indicated that HOXA3 acted upstream of these genes and a critical regulator of these key genes in the development of endoderm of the third pharyngeal pouch.

Next, we performed GO functional enrichment on the differential genes using transcriptome sequencing data which were obtained before and after HOXA3 inhibition in 3PPE, and found enrichments of biological processes such as cell population proliferation, animal organ development, and cell migration (**Figure 5K**), and also found that HOXA3 was involved in regulating migration (**Figure 5L**) and proliferation (**Figure 5M**) of epithelial cells by GSEA analysis. To this end, healing assays and transwell migration assays were performed to determine the effect of HOXA3 inhibition on the migration of 3PPE. Notably, the migratory ability of 3PPE cells was enhanced after HOXA3 inhibition with siRNA for 24 hours in healing assays (**Figure 5N**), and the number of cells that migrated through transwells 72 hours after the culture was much higher than those in the NC group in transwell migration assays (**Supplementary Figure 6E**). The Heat map results also showed significant upregulation of cell migration related genes after HOXA3 inhibition with siRNA (**Figure 5O**). CTHRC1 (26, 27) and INSL3 (28, 29) promote cell migration, the results of RT-qPCR assay exhibited the increased migratory capacity of cells by increasing the expressions of CTHRC1 and INSL3 following HOXA3 inhibition (**Figure 5P**). Moreover, we further performed cell cycle assay on 3PPE 48 hours after the transduction with siRNAs, and found that the inhibition of HOXA3 led to G1 arrest in the cell cycle (**Supplementary Figure 6F**), and the expression levels of proliferation related genes PCNA and Cdc25C were also significantly downregulated (**Supplementary Figure 6G**). Additionally, we also evaluated the role of HOXA3 in cell proliferation of 3PPE. Interestingly, using cell counting kit-8 assay, we found that the reduction of HOXA3 inhibited the proliferation of 3PPE cells (**Figure 5Q**), qRT-PCR results further verified that the expression levels of MCM2 and CDC45L as well as c-Myc, which are related to cell proliferation, were also significantly downregulated (**Figure 5R**). Taken together, our results revealed that the up-regulation of HOXA3 promoted the differentiation and proliferation and inhibited the migration, on the contrary, the down-regulation of HOXA3 enhanced the migration and suppressed the differentiation and proliferation, thus, these findings indicated that HOXA3 functioned as the on-off switch to regulate the differentiation and proliferation as well as the migration during the development of hESC-derived 3PPE.

### HOXA3 regulated Wnt signaling and EPHB2 in 3PPE

**Figure 6:**
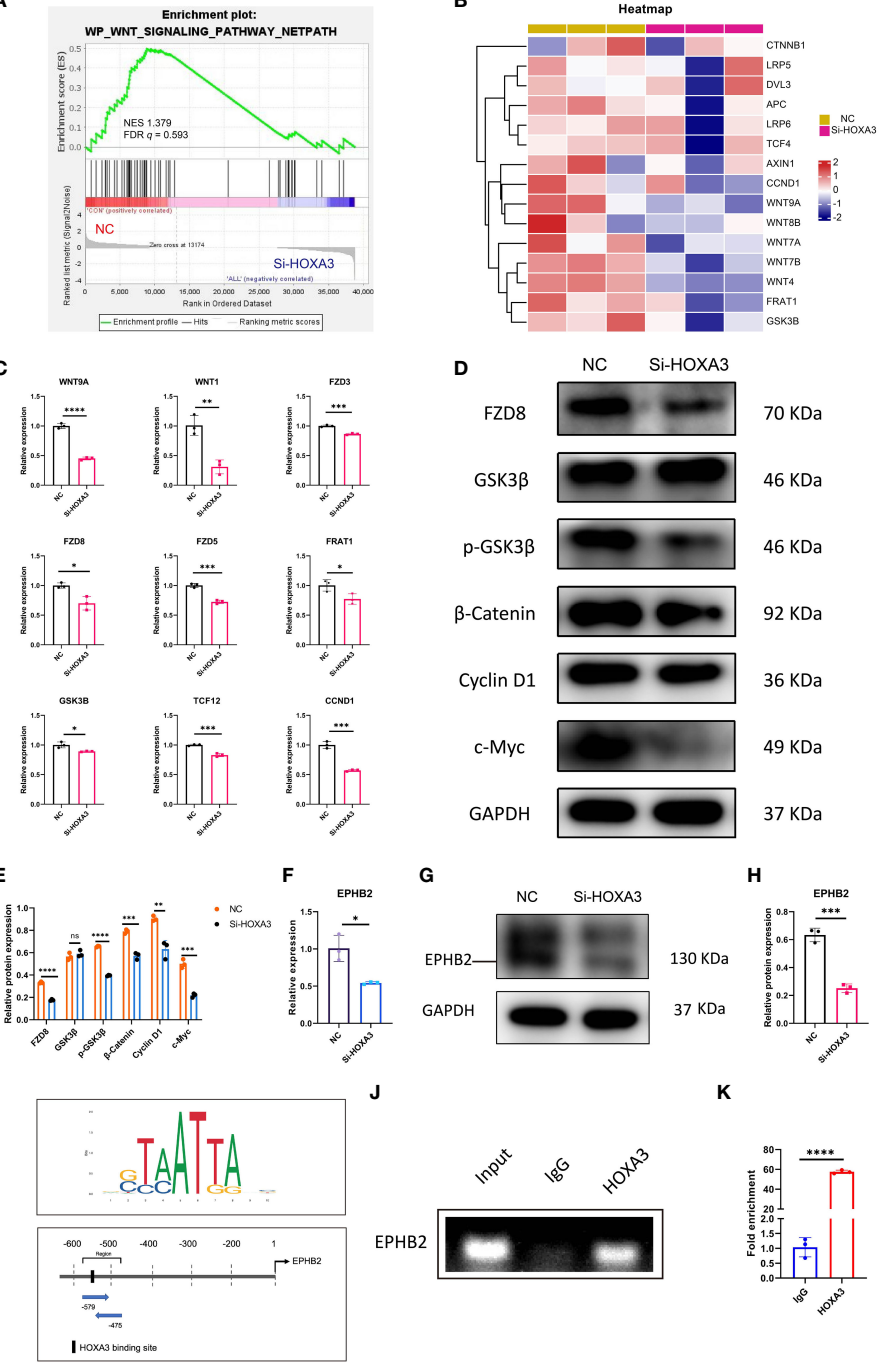
Wnt/β-catenin signaling and EPHB2 were the downstream effector of HOXA3 in 3PPE. **(A)** GSEA analysis showed that the genes regulating Wnt signaling pathway were down-regulated after HOXA3 inhibition with a normalized enrichment score of 1.379 (FDR *q* value of 0.593). **(B)** Heat map exhibited the transcriptional changes of genes associated with WNT signaling pathway in cells of 3PPE after the treatment with HOXA3 siRNA. **(C)** The expression changes of genes associated with WNT signaling pathway were assessed by qRT-PCR in cells treated with HOXA3 siRNA. **(D, E)** Western blot was used to evaluate the protein expressions of FZD8, GSK3β, p-GSK3β, β-Catenin, Cyclin D1 and c-Myc in cells treated with HOXA3 siRNA **(D)**, and the protein levels were normalized to those of housekeeping gene GAPDH **(E)**. **(F)** The expression changes of EPHB2 was assessed by qRT-PCR 48 hours after the treatment of cells with HOXA3 siRNA. **(G, H)** Western blot was used to evaluate the protein expression of EPHB2 in cells treated with HOXA3 siRNA **(G)**, and the protein levels were normalized to those of housekeeping gene GAPDH **(H)**. **(I)** Upstream binding sites of HOXA3 in the promoter of EPHB2 were predicted. Sequence logo of HOXA3 (Upper panel). Possible binding sites of HOXA3 in the location and area of EPHB2 promoter (Bottom panel). **(J)** PCR was performed in ChIP assays to amplify DNA samples precipitated with HOXA3 antibodies using specific primers to evaluate the recruitment of HOXA3 on EPHB2 promoter. Normal rabbit immunoglobulin G (IgG) or no antibody was used as negative controls. **(K)** Quantitative ChIP-qPCR assay was performed with the 3PPE cells using IgG and HOXA3 antibody followed by amplification of DNA fragments of EPHB2 promoter. Data was normalized to input fraction and the results were calibrated to that of IgG which was set 1. Values were presented in mean ± SD. *p < 0.05, **p < 0.01, ***p < 0.001, ****p < 0.0001. qRT-PCR data were shown normalised to the NC condition. n = 3 independent biological repeats.

The Wnt signaling pathway is inextricably linked to cell migration (30, 31), proliferation (32, 33), as well as differentiation (34, 35), thus, we next explored whether HOXA3 had a direct relationship with Wnt signaling pathway. As expected, we found that Wnt signaling pathway was significantly downregulated upon HOXA3 inhibition in GSEA analysis (**Figure 6A**), and these significant repressions included CTNNB1, LRP5, DVL3, APC, LRP6, TCF4, AXIN1, CCND1, WNT9A, WNT8B, WNT7A, WNT7B, WNT4, FRAT1, as well as GSK3β in heat map (**Figure 6B**). In order to clarify whether the Wnt pathway was influenced by HOXA3, we examined Wnt signaling related genes at both the protein and mRNA expression levels. qRT-PCR analysis showed that the knockdown of HOXA3 induced significant repressions of a large number of Wnt signaling related genes, including WNT9A, WNT1, FZD3, FZD8, FZD5, FRAT1, GSK3B, TCF12, CNND1 (**Figure 6C**). In addition, Western blotting was performed to further confirm that protein expression levels of FZD8, p-GSK3β, β-catenin, Cyclin D1 and c-Myc were obviously decreased in 3PPE after the treatment with HOXA3 siRNA (**Figures 6D, E**). EPHB2 is expressed in intestinal epithelial cells, and EPHB receptor is the target gene of Wnt signal (36), and EPHB receptors are thought to be positive regulators of proliferation in colon crypts (32). Thus, we further explored whether HOXA3 regulated EPHB2. First, we detected the relative expression of EPHB2 by qRT-PCR after inhibiting HOXA3, and found that EPHB2 was down-regulated (**Figure 6F**), and this down-regulation was verified at protein expression level by Western blot (**Figures 6G, H**). Importantly, we found that HOXA3 had a binding site upstream of the promoter of EPHB2 through JASPAR transcription factor motif database, and relative score is greater than 0.8 (**Figure 6I**; **Supplementary Figure 7**; **Supplementary Table 2**). Next, we determined the mediator which linked HOXA3 and Wnt signaling pathway by identifying the direct target of HOXA3, then chromatin immunoprecipitation (ChIP) assays were performed to use different primers that encompassed the EPHB2 promoter region for detecting the interaction between HOXA3 and EPHB2 promoter in 3PPE cells, and the results of ChIP PCR analysis (**Figure 6J**) and Chip-qPCR (**Figure 6K**) using primary antibody against anti-HOXA3 showed that DNA fragments treated with anti-HOXA3 antibody were significantly higher than those in IgG group, and that HOXA3 physically interacted with EPHB2, indicating EPHB2 as the target of HOXA3.

### HOXA3 regulated EPHB2-mediated Wnt signaling which in turn regulated migration and proliferation of 3PPE

**Figure 7:**
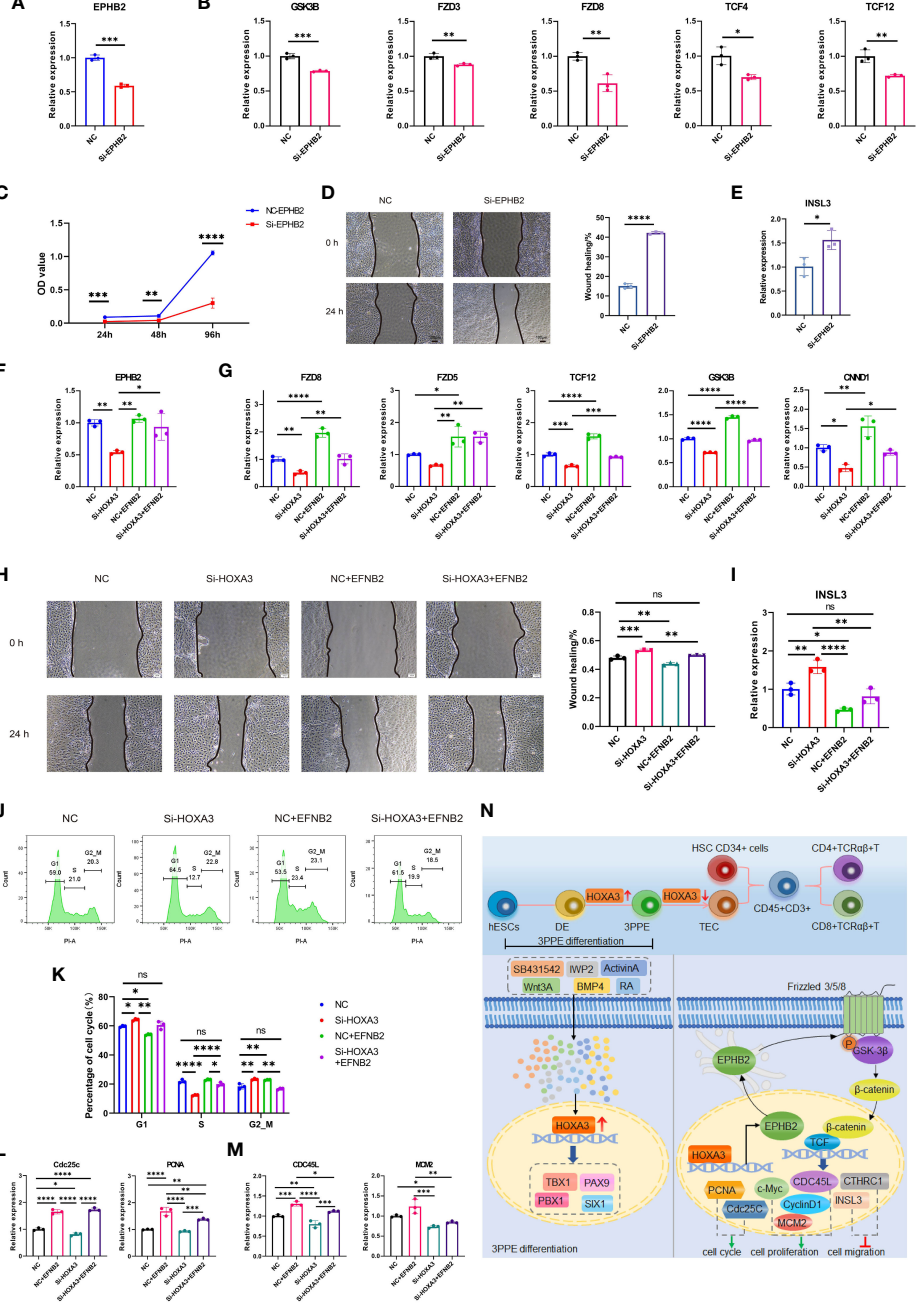
HOXA3 regulated EPHB2-mediated Wnt signaling which in turn regulated migration and proliferation of 3PPE. **(A)** The expression of EPHB2 gene was assessed by qRT-PCR after the treatment with EPHB2 siRNA. **(B)** The expression changes of genes associated with WNT signaling pathway were assessed by qRT-PCR in cells treated with EPHB2 siRNA. **(C)** Cell proliferation assay was determined by CKK8 at 24, 48 and 96 hours after the treatment of cells with EPHB2 siRNA. **(D)** Representative images (Left panel) and quantitation (Right panel) of wound-healing assay for the measurement of cell migration 24 hours after the treatment with EPHB2 siRNA. **(E)** The expression change of INSL3 was assessed by qRT-PCR in cells treated with EPHB2 siRNA. **(F)** qRT-PCR analysis for expression of EPHB2 in 3PPE treated with Si-HOXA3, NC plus EFNB2 or Si-HOXA3 plus EFNB2. **(G)** qRT-PCR analysis for expression of FZD8, FZD5, TCF12, GSK3B and CNND1 in 3PPE treated with Si-HOXA3, NC plus EFNB2 or Si-HOXA3 plus EFNB2. **(H)** Representative images (Left panel) and quantification (Right panel) of Wound healing assays for the measurement of migration of 3PPE 24 hours after the treatment with Si-HOXA3, NC plus EFNB2 or Si-HOXA3 plus EFNB2. **(I)** qRT-PCR analysis for expression of INSL3 in 3PPE treated with Si-HOXA3, NC plus EFNB2 or Si-HOXA3 plus EFNB2. **(J, K)** Cell cycle distribution of cells was determined by flow cytometry with propidium iodide staining 48 hours after the treatment with SiHOXA3, NC plus EFNB2 or SiHOXA3 plus EFNB2. **(L, M)** The expression changes of cell cycle markers Cdc25c and PCNA **(L)** and cell proliferation markers **(M)** were determined by qRT-PCR in cells after the treatment with Si-HOXA3, NC plus EFNB2 or Si-HOXA3 plus EFNB2. **(N)** Schematic diagram illustrating that HOXA3 regulated the development of hESC-derived 3PPE. Values were presented in mean ± SD. *p < 0.05, **p < 0.01, ***p < 0.001, ****p < 0.0001. qRT-PCR data were shown normalised to the NC condition. n = 3 independent biological repeats.

In order to clarify whether HOXA3 regulated Wnt signal through EPHB2, we designed rescue experiments to verify that EPHB2 served as vital downstream target gene of HOXA3. First, we used EPHB2 siRNA to determine the effect of EPHB2 on Wnt signaling pathway. To this end, we used siRNA against EPHB2 during the differentiation of hESCs into 3PPE at day 7, and we found that the expression of EPHB2 was significantly reduced (**Figure 7A**). Next, we further evaluated the consequence by qRT-PCR, and found that the repression of EPHB2 led to downregulation of GSK3B, FZD3, FZD8, TCF4, TCF12 (**Figure 7B**). Moreover, we further performed cell cycle assay on 3PPE cells 48 hours after siRNA transduction, and the results showed that the inhibition of EPHB2 led to G1 arrest in the cell cycle (**Supplementary Figures 8A, B**), and the expression levels of proliferation related genes PCNA and Cdc25C were also significantly downregulated (**Supplementary Figures 8C, D**). Additionally, we also evaluated the role of EPHB2 in the proliferation of 3PPE cells. Interestingly, using cell counting kit-8 assay, we found that the reduction of EPHB2 inhibited the proliferation of 3PPE cells (**Figure 7C**). Thus, healing assays were performed to determine the effect of EPHB2 inhibition on the migration of 3PPE cells. Notably, the migratory ability of 3PPE cells was enhanced after EPHB2 inhibition with siRNA for 24 hours in healing assays (**Figure 7D**), and the results of RT-qPCR assay exhibited the increased migratory capacity of cells by increasing the expressions of INSL3 following EPHB2 inhibition (**Figure 7E**). In order to determine targeting specificity of HOXA3 to the mediator of Wnt signaling pathway, the rescue experiments were performed. During the differentiation process of endodermal cells of 3PP, we first employed different concentrations of activator EFNB2 of EPHB2 at 100ng/mL, 200ng/mL and 500ng/mL to evaluate the enhancement of EPHB2 expression. The results showed that the addition of 200 ng/mL EFNB2 significantly increased the expression of EPHB2 and CNND1 in 3PPE cells (**Supplementary Figures 8E, F**). Next, we conducted qRT-PCR analysis for examining the rescue effect of activator EFNB2 of EPHB2 on the expressions of EPHB2 (**Figure 7F**) and markers of Wnt signaling pathway (FZD8, FZD5, TCF12, GSK3B, and CNND1) after the inhibition of HOXA3 with siRNA (**Figure 7G**). Consistently, HOXA3 siRNA profoundly down-regulated the expressions of these genes, however, the expression of these genes was increased or rescued by the addition of activator EFNB2 of EPHB2 (**Figures 7F, G**). Thus, these results demonstrated that HOXA3 regulated WNT pathway specifically through EPHB2. In order to further verify this mechanism, wounding healing assays were performed, and we observed a faster wound healing in the HOXA3 siRNA-treated group versus the NC group, indicating that the HOXA3 inhibition enhanced the migration of the cells (**Figure 7H**). Of note, the addition of EPHB2 activator EFNB2 protein was capable to markedly reduce cell migration which was promoted by HOXA3 inhibition (**Figure 7H**). Meanwhile, we also found that the migration gene INSL3 was significantly upregulated in response to HOXA3 inhibition, but was suppressed by the addition of EFNB2 (**Figure 7I**), indicating that EFNB2 prevented the cell migration under HOXA3 or EPHB2 inhibition. In addition to this phenomenon, cell cycle assays also were further performed using flow cytometry, and the results illustrated that EFNB2, an activator of EPHB2, was able to increase S phase in the cell cycle after the treatment with HOXA3 siRNA (**Figures 7J, K**), indicating that the extra expression of EFNB2 promoted the cell division (growth) after the prevention of the migration. Moreover, we conducted qRT-PCR analysis for examining the effects of EPHB2 on the expression of mitotic cell cycle markers PCNA and Cdc25c and cell proliferation markers CDC45L and MCM2. However, the addition of EFNB2 increased the expression cell cycle markers PCNA and Cdc25c (**Figure 7L**) and cell proliferation markers CDC45L and MCM2 (**Figure 7M**) in the cells of 3PP after the treatment with HOXA3 siRNA, further indicating that the extra expression of EFNB2 promoted the cell proliferation coupled with the inhibition of the migration under HOXA3 or EPHB2 inhibition. Taken together, these results demonstrated that the upregulation of HOXA3 by growth factors and small molecules induced the differentiation of 3PPE from hESCs and the expression of EPHB2 which activated Wnt signaling pathway to promote the cell proliferation of 3PPE, then the downregulation of HOXA3 switched its function to promote the cell migration, and suppressing the cell proliferation also through the inhibition of EPHB2-mediated Wnt pathway (**Figure 7N**).

## Discussion

The thymus and parathyroid glands develop from the thymus or parathyroid primordium, which is formed from the endoderm of 3PP. The 3PP, which is formed by evagination of the endoderm-derived epithelial layer from the gut tube around embryonic day (E) 9.5–10.5 in C57BL/6 mice (5). In 3PP, the epithelium is ensheathed by neural crest-derived mesenchymal cells (37), between which they direct the commitment of 3PPE by secreting soluble factors, extracellular matrix, and other interactions. For example, BMP4 signaling plays a crucial role during the early formation of thymus and parathyroid glands (38). In this study, we developed a novel protocol for differentiating hESCs into 3PPE in a manner, and found that culture condition with DMEM/F-12 supplemented with WNT3A at the endodermal stage was more favorable for the development of 3PPE at later stages. In this differentiation condition, we could obtain nearly 100% purity for PAX9^+^EpCAM^+^ cells as well as SIX1^+^EpCAM^+^ cells. This was sufficient to show the establishment of an efficient differentiation approach for the endoderm of 3PP from hESCs.

The 3PP will eventually develop into the parathyroid and thymus glands, and in this process, the 3PPE cells undergo an extremely complex process. At mouse embryonic E11.5, the transcription factor Foxn1 is a TEC functional gene that starts to be expressed in the caudal ventral part of the epithelial primordium (37). Genetic defects in FOXN1 transcription factor result in thymus hypoplasia and severe immunodeficiency in humans (39). Transcriptional regulatory networks are central regulatory mechanisms controlling organ identity, patterning, and differentiation. At present, several key transcription factors have been identified in the thymus that are critical for various aspects of thymus organogenesis and TEC differentiation. The thymus gland is formed during the embryogenesis by the differentiation of 3PPE, and in the formation of thymic epithelia as development proceeds, which include cortical TECs and medullary TECs. There are now well-known transcription factors that mediate various stages of thymic organ development, including a HOXA3 dependent cascade of initial fate specification, Foxn1 expressed by early or/and later TECs, and NF kappaB expressed by mTECs (40).

We induced TECs from hESC-derived 3PPE employing an optimal differentiation system, which included DMEM-F12 medium supplemented with a variety of chemical small molecules and cytokines as well as growth factors, such as RA, heparin, SB431542, FGF8, FGF10, WNT3A, BMP4, and ascorbic acid. Mature TECs that are MHC-II^+^EpCAM^+^ cells could be differentiated from hESCs under our condition. More importantly, not only cortical TECs but also large number of medullary TECs were included in hESC-derived epithelium. Concomitantly, we established a novel co-culture of hESC-derived TECs with human HSCs to demonstrate that those functional TECs supported T cell development.

In vertebrates, HOXA3 (homeobox A3), one of the family genes encoding the class of transcription factors called homeobox genes are found in clusters named A, B, C, and D on four separate chromosomes. The expression of these proteins is spatially and temporally regulated during the embryonic development. This gene is part of the A cluster on chromosome 7 and encodes a DNA-binding transcription factor which regulate gene expression, morphogenesis, and differentiation (41). HOXA3 deficient mice have severe defects not only in pharyngeal organ development, including thyroid hypoplasia and insufficiency, and parathyroid insufficiency, but also in endoderm and laryngeal cartilage as well as cranial nerves (41). HOXA3 is required for tissue and organ differentiation of endodermal cells (tracheal epithelium, thymus and parathyroid glands) and contributes to organ migration and morphogenesis of NCC (5). In addition, thymus/parathyroid primordium not only appear hypoplastic at the time of formation, but also cause thymic ectopy due to delayed separation of thymic*/*parathyroid primordia from the pharynx in HOXA3 (+/-) Pax1 (-/-) compound mutants (42). HOXA3 null mutants have altered locations and timing of key region markers within the pocket, including Tbx1, BMP4, and FGF8, and a single tissue-specific HOXA3 deletion results in a small ectopic thymus. In the endoderm, HOXA3 temporally regulates the initiation of the thymic program and is required for parathyroid differentiation in a cell autonomous manner (2). The mechanisms that control various morphogenetic events during early thymus organogenesis remain largely unknown, although a few genes influencing these events have been identified so far. The transcription factors HOXA3 and Pax1/9 functioning in specific pathways are required for the proper separation and/or migration of the developing thymus and parathyroid primordia from the pharynx (10, 42, 43). Thus, at present, it appears that, although HOXA3 functions very robustly and its absence leads to very dramatical influence on embryonic organ development, little is known about how HOXA3 acts on cells to function. To this regard, we used siRNA to knockdown HOXA3 in 3PPE cells for probing the function of HOXA3 and potential mechanism, and found that the repression of HOXA3 indeed has a crucial effect on the development of 3PPE. Our results indicated that HOXA3 regulated key genes of 3PPE, such as SIX1, PAX9, Tbx1, Pbx1 and BMP4 for the development of 3PPE, and also had functions not only in regulating cell migration, but also in playing an irreplaceable role in cell cycle as well as cell proliferation. Cell migration was promoted by the down-regulation of HOXA3, echoing the dynamic changes of the HOXA3 gene during mouse embryonic development and the morphological changes in the gradual migratory division of 3PP into thymic primordia and parathyroid primordia. During this process, the proliferation of the cells was decreased by the cell cycle arrest at G1 phase, and this decrease was essential for the cell migration, and HOXA3 functioned as the on-off switch contributed to this dynamic change. The maturation of T cells is results from the interactions between TECs and thymocytes, and mice with the HOXA3^+/-^Pax1^-/-^ compound mutation exhibited more severe thymic defects compared to Pax1^-/-^ single mutants and had fewer MHC class II^+^ epithelial cells, thus affecting early thymocyte maturation as well as a drastic reduction of CD4^+^CD8^+^ DP cells (44). Thus, it follows that HOXA3 plays a pivotal role in the differentiation, proliferation of the endoderm of the third pharyngeal pouch, migration of the thymus and parathyroid primordium, and thymus colonization of the upper chamber of the heart in conjunction with functions in T cell maturation.

Wnt signaling pathway is an evolutionarily conserved cell-cell communication system that is important for stem cell renewal, cell proliferation and differentiation during both the embryogenesis and adult tissue homeostasis (45). Hox transcription factor Hoxb8a is essential for proper migration and acts downstream of Wnt signaling to regulate the spatial expression of both chemokine receptors (46). EPHB2, belongs to the Eph receptor family of receptor tyrosine kinase transmembrane glycoproteins, these receptors bind ligands called ephrins and are involved in diverse cellular processes, including motility, division, and differentiation (47). In particular, EPHB2 expression is most prominent to promote cell proliferation in the intestinal epithelium (48, 49). The ligands of Eph kinases are ephrins (EFNs), which are cell surface proteins (50). The EFNB subfamily consists of three members (EFNB1 to 3), and they are transmembrane proteins (51, 52). Although they are ligands, EFNs, especially EFNB subfamily members, can reversely transduce signals into cells (52, 53). The interaction between Ephs and EFNs is not very strict, one Eph can bind to several different EFNs and vice versa (50). For example, EFNB2 plays a role in immune regulation, which is a ligand of several EphB kinases, including, but not restricted to, EphB6 (50). EFNB2 mRNA was expressed in the cortex of the thymus and white pulp of the spleen (50). At the protein level, it was expressed in T cells and monocytes/macrophages, but not in B cells (50). Previous studies have demonstrated that EphB2 and EphB3 kinases, receptors for ephrinB1 and ephrinB2, are necessary for the proper organization of thymic epithelium (54, 55). Using EphB2 and/or EphB3 knockout mice, an abnormal thymic development was shown to mainly affect to the epithelial components, including the cortex/medulla distribution, the morphology of TECs and the expression of different epithelial-specific markers (56). It has demonstrated that the lack of ephrinB1 and/or ephrinB2, either on thymocytes or on TECs, alters the cell intermingling processes necessary for thymus organization, and affects cortical TEC subpopulations (56). Eph/ephrins are extensively expressed in the thymus and seem to be involved in the colonization of lymphoid progenitor cells and their migration throughout the thymic parenchyma necessary to provide an adequate topological location of developing thymocytes in the epithelial network that ensures their correct differentiation (57). In addition, EphB2 and EphB3 play a cell-autonomous role in regulating the transitions of double-negative cells to double-positive cells and of double-positive thymocytes to single-positive thymocytes and the lack of these molecules or their ligands ephrin B1 and ephrin B2 induces profound the alterations of the maturation TECs and the arrangement of epithelial network (57).

Furthermore, Wnt/β-Catenin and their downstream ephrinB/EPHB signaling are critical for the proliferation and localization of intestinal epithelial cells during the migration (36). EPHB2 acts through driving Src/Akt/GSK3β/β-Catenin signaling cascades and regulates cancer stemness and drug resistance (58). *In vivo*, EFNB2 expressed by neural crest cells/thymic mesenchyme is required for the migration of the thymic primordium (59). Notably, in the absence of ephrin-B2 expression on thymic NC-derived mesenchyme, the thymus remains in the cervical area instead of migrating into the thoracic cavity, and the deletion of Ephrin-B2 does not disrupt the separation of the thymus/parathyroid rudiment from the third pharyngeal pouch (59). In addition to that, the expression of Hoxa3 appears normal in mice with Ephrin-B2–deficient NCCs (59).

In terms of mechanism, we presented for the first time that HOXA3 could regulate the differentiation of the inner coordination cells of 3PP through the downstream signaling pathway of Wnt signaling. Our findings demonstrated that the downregulation of Wnt signaling in 3PPE cells was results of repressing HOXA3. By ChIP assay, our results showed that HOXA3 directly interacted with EPHB2, thereby indicating its role in regulating Wnt signaling pathway through EPHB2. Importantly, EPHB2 acted on the positive or negative indicator of the on-off switch of HOXA3 in regulating the migration and proliferation of 3PPE cells during the crosstalk among HOXA3, EPHB2 and WNT pathway, the expression of EPHB2 indicated that the up-regulation of HOXA3 positively regulated WNT pathway for promoting cell proliferation, and the repression of EPHB2 exhibited that the down-regulation of HOXA3 negatively regulated (down-regulated) WNT pathway for enhancing cell migration.

In conclusion, we established a highly efficient approach to differentiate 3PPE from hESCs and validated its function by differentiating into TECs with the capacity to promote the maturation of T cells, and our results exhibited that HOXA3 played function as the on-off switch to regulate the differentiation, migration, and proliferation of 3PPE. More importantly, our finding further demonstrated that HOXA3 played function through EPHB2 to regulate Wnt signaling pathway to maintain the developmental commitment and function of 3PPE.

## Data availability statement

The datasets presented in this study can be found in online repositories. The names of the repository/repositories and accession number(s) can be found below: GSE237751 (GEO-https://www.ncbi.nlm.nih.gov/geo/query/acc.cgi?acc=GSE237751).

## Ethics statement

The studies involving humans were approved by Ethics Committee of Guangzhou First People’s Hospital (Approval No K-2021-008-02). The studies were conducted in accordance with the local legislation and institutional requirements. The participants provided their written informed consent to participate in this study. Ethical approval was not required for the studies on animals in accordance with the local legislation and institutional requirements because only commercially available established cell lines were used.

## Author contributions

YD and YF conceived the project and designed the experiments. YF and XZ performed experiments, analyzed data and performed bioinformatics. HW cultured and maintained hESCs. PZ, SL, TG, HS, YL, HC and JX contributed expertise. YF and YD wrote and revised the manuscript. YD and JX provided financial supports. All authors contributed to the article and approved the submitted version.
