# Supplementary material for: HOXA3 functions as the on-off switch to regulate the development of hESC-derived third pharyngeal pouch endoderm through EPHB2-mediated Wnt pathway
Source: Front Immunol. 2024 Jan 8;14:1258074. doi: 10.3389/fimmu.2023.1258074 (PMC10800530; doi:10.3389/fimmu.2023.1258074)
Supplement: Supplementary file 1 [file DataSheet_1.pdf]

Supplementary material

HOXA3 functions as the on-off switch to regulate the development of hESC-derived third pharyngeal pouch endoderm through EPHB2-mediated Wnt pathway

Yingjie Fu#, Xueyan Zhang#, Haibin Wu, Pingping Zhang, Shoupei Liu, Tingting Guo, Huanhuan Shan, Yan Liang, Honglin Chen, Jinghe Xie*, Yuyou Duan*

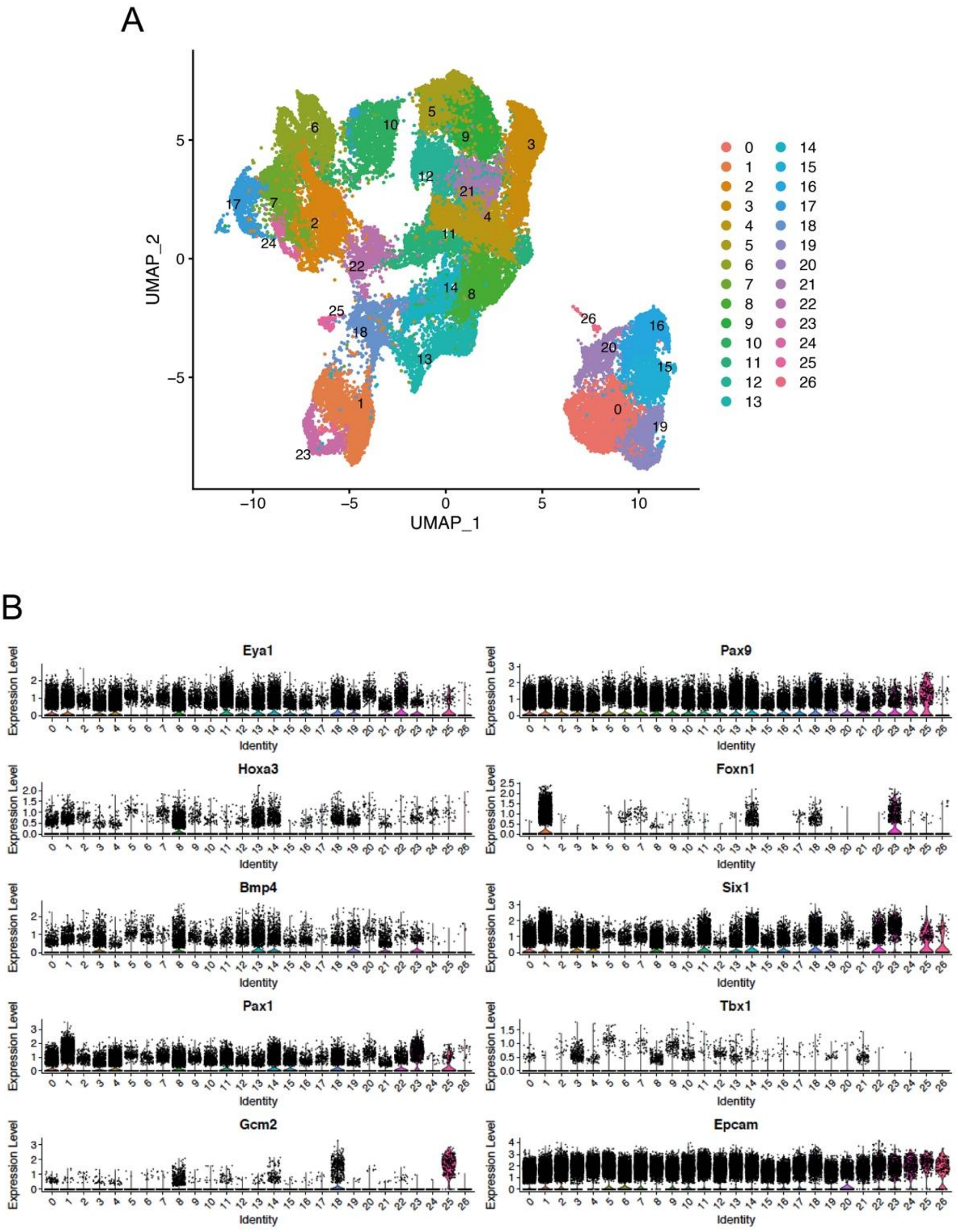
A
UMAP_1
UMAP_2
B
Eya1
Pax9
Hoxa3
Foxn1
Bmp4
Six1
Pax1
Tbx1
Gcm2
Epcam
Expression Level
Identity

Supplementary Figure 1. Development of the pharyngeal pouch in a mouse embryo by single-cell transcriptome sequencing

(A) Unsupervised UMAP analysis of Pax9^{+}Epcam^{+} mouse embryos cell clusters. mouse embryos cells were pooled from two E9.5, three E10.5, three E11.5 and two E12.5 samples. (B) Violin plot showed the expression of related genes in the third pharyngeal pouch, thymus and parathyroid glands in 27 cell clusters.

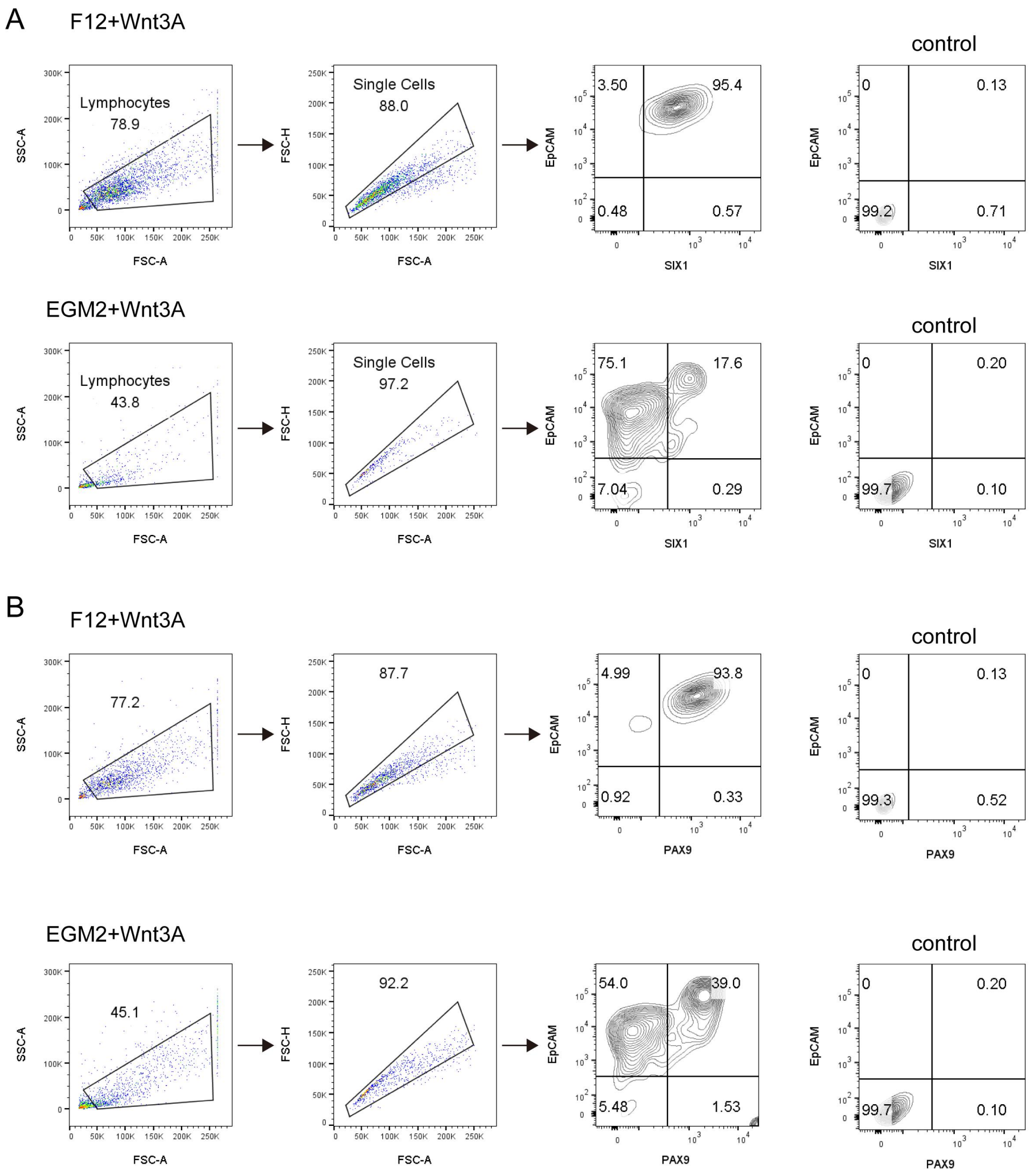
A
F12+Wnt3A
Lymphocytes
78.9
Single Cells
88.0
3.50
95.4
0.48
0.57
control
0
0.13
99.2
0.71
SSC-A
FSC-A
FSC-H
EpCAM
SIX1
EGM2+Wnt3A
Lymphocytes
43.8
Single Cells
97.2
75.1
17.6
7.04
0.29
control
0
0.20
99.7
0.10
B
F12+Wnt3A
77.2
87.7
4.99
93.8
0.92
0.33
control
0
0.13
99.3
0.52
PAX9
EGM2+Wnt3A
45.1
92.2
54.0
39.0
5.48
1.53
control
0
0.20
99.7
0.10

Supplementary Figure 2. Differentiation of 3PPE from hESCs

(A) The proportions of SIX1 and EpCAM double positive cells were measured by flow cytometry at days 9 after the differentiation. **(B)** The proportions of PAX9 and EpCAM double positive cells were measured by flow cytometry at days 9 after the differentiation. The control was shared in A and B due to the same sample data source and using the same fluorescence channel.

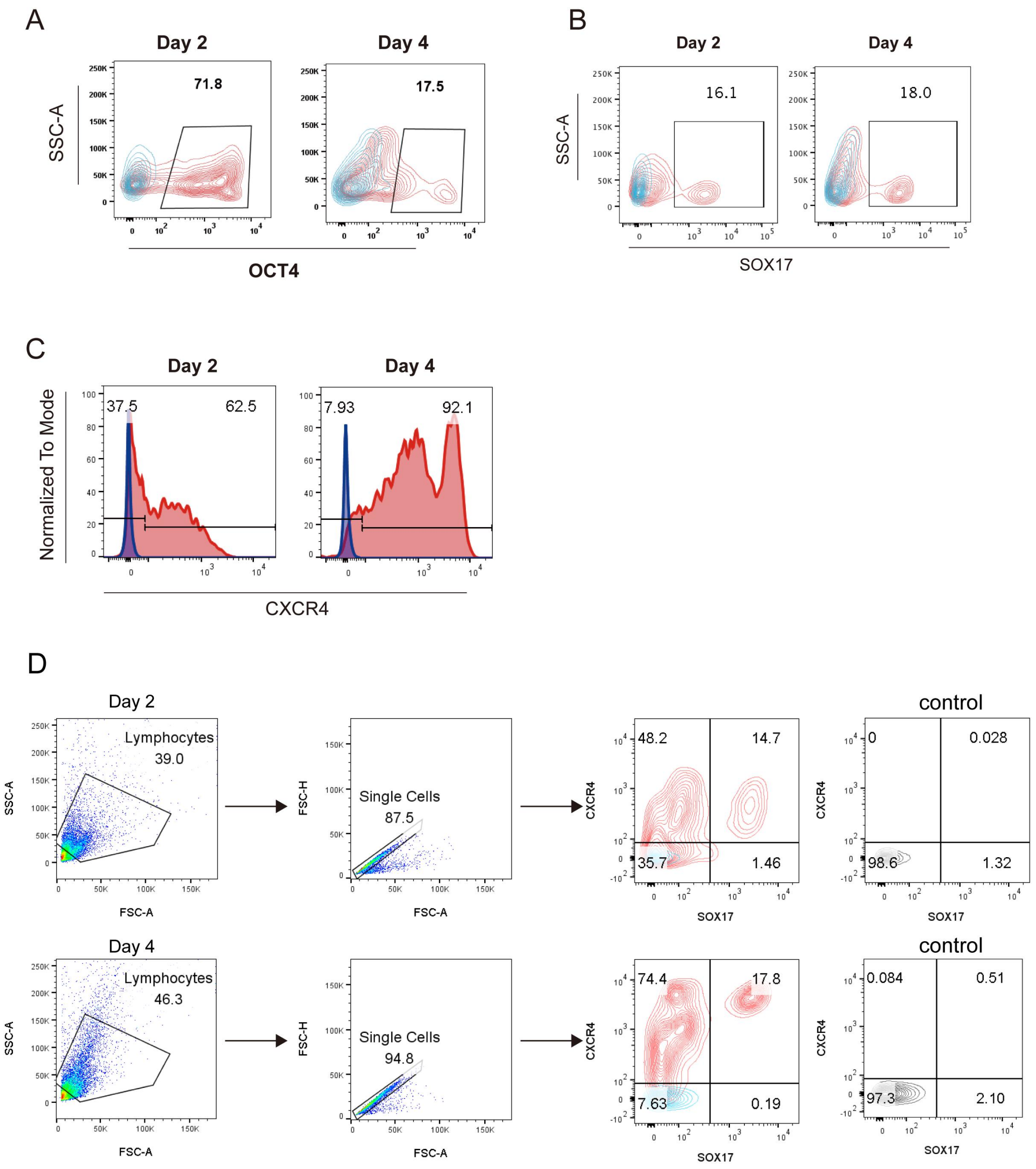
A
Day 2
Day 4
SSC-A
71.8
17.5
OCT4
B
Day 2
Day 4
SSC-A
16.1
18.0
SOX17
C
Day 2
Day 4
Normalized To Mode
37.5
62.5
7.93
92.1
CXCR4
D
Day 2
Lymphocytes
39.0
SSC-A
FSC-A
FSC-H
Single Cells
87.5
CXCR4
SOX17
48.2
14.7
35.7
1.46
control
0
0.028
98.6
1.32
Day 4
Lymphocytes
46.3
Single Cells
94.8
74.4
17.8
7.63
0.19
control
0.084
0.51
97.3
2.10

Supplementary Figure 3. Identification and characterization of endodermal cells and 3PPE derived from hESCs

(A) The population of pluripotent marker $OCT4^+$ cells were measured by flow cytometry at days 2 and 4. **(B)** The proportions of SOX17 positive cells were measured by flow cytometry at days 2 and 4 after the differentiation. **(C)** The proportions of CXCR4 positive cells were measured by flow cytometry at days 2 and 4 after the differentiation. **(D)** The proportions of CXCR4 and SOX17 double positive cells were measured by flow cytometry at days 2 and 4 after the differentiation.

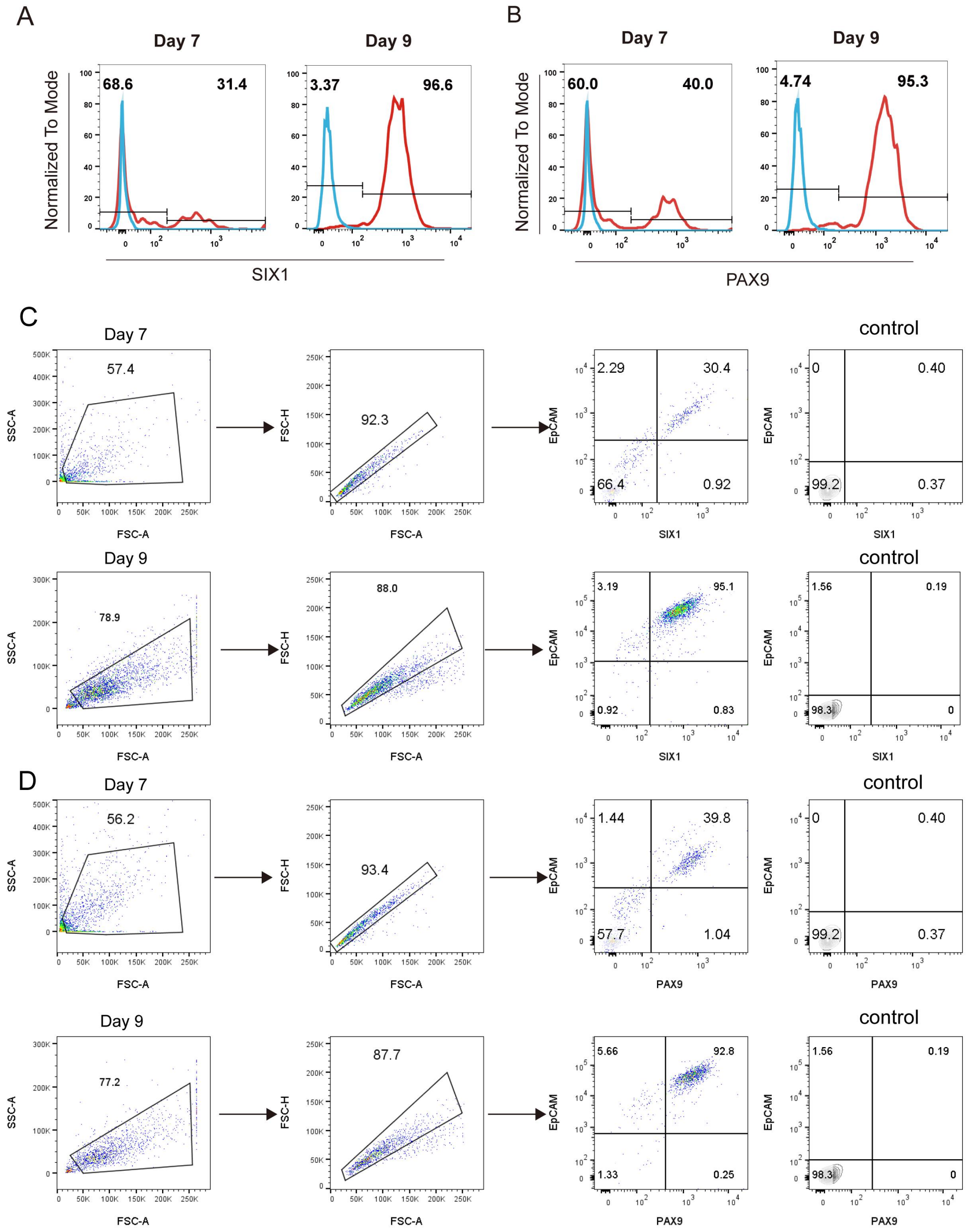
A
Day 7
Day 9
Normalized To Mode
68.6
31.4
3.37
96.6
SIX1
B
Day 7
Day 9
60.0
40.0
4.74
95.3
PAX9
C
Day 7
57.4
92.3
2.29
30.4
66.4
0.92
control
0
0.40
99.2
0.37
SSC-A
FSC-A
FSC-H
EpCAM
SIX1
Day 9
78.9
88.0
3.19
95.1
0.92
0.83
1.56
0.19
98.3
0
D
Day 7
56.2
93.4
1.44
39.8
57.7
1.04
PAX9
Day 9
77.2
87.7
5.66
92.8
1.33
0.25

Supplementary Figure 4. Identification and characterization of endodermal cells and 3PPE derived from hESCs

(A) The proportions of SIX1 positive cells were measured by flow cytometry at days 7 and 9 after the differentiation. **(B)** The positive proportions of cells for PAX9 were measured by flow cytometry at days 7 and 9 after the differentiation. **(C)** The proportions of SIX1 and EpCAM double positive cells were measured by flow cytometry at days 7 and 9 after the differentiation. **(D)** The double positive proportion of cells for PAX9 and EpCAM were measured by flow cytometry at days 7 and 9 after the differentiation. The control was shared in C and D due to the same sample data source and using the same fluorescence channel.

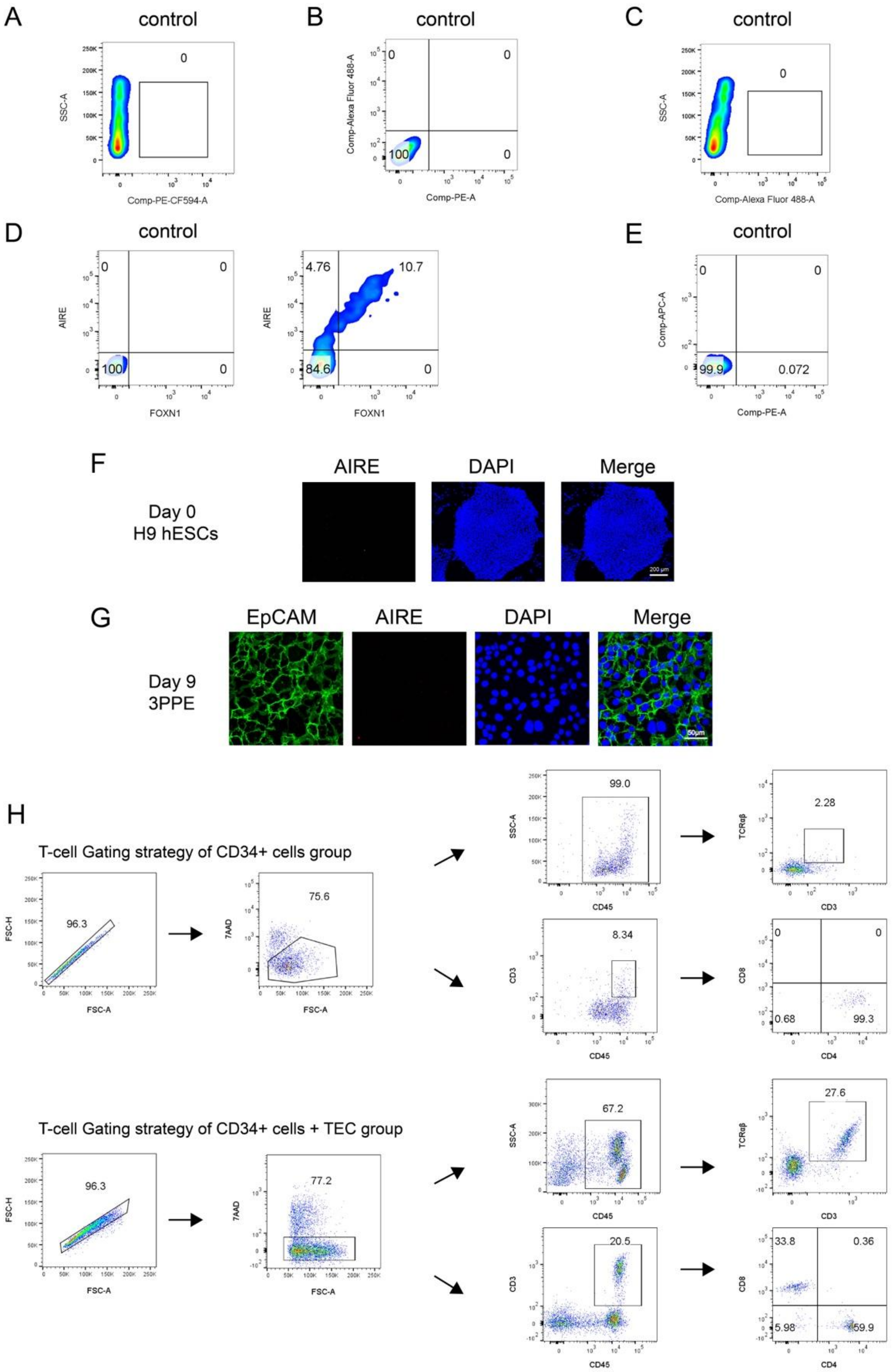

A
control
0
SSC-A
Comp-PE-CF594-A
B
control
0
0
100
0
Comp-Alexa Fluor 488-A
Comp-PE-A
C
control
0
SSC-A
Comp-Alexa Fluor 488-A
D
control
0
0
100
0
AIRE
FOXN1
4.76
10.7
84.6
0
E
control
0
0
99.9
0.072
Comp-APC-A
Comp-PE-A
F
AIRE
DAPI
Merge
Day 0
H9 hESCs
200 μm
G
EpCAM
AIRE
DAPI
Merge
Day 9
3PPE
50μm
H
T-cell Gating strategy of CD34+ cells group
96.3
FSC-H
FSC-A
75.6
7AAD
FSC-A
99.0
SSC-A
CD45
2.28
TCRαβ
CD3
8.34
CD3
CD45
0
0
0.68
99.3
CD8
CD4
T-cell Gating strategy of CD34+ cells + TEC group
96.3
FSC-H
FSC-A
77.2
7AAD
FSC-A
67.2
SSC-A
CD45
27.6
TCRαβ
CD3
20.5
CD3
CD45
33.8
0.36
5.98
59.9
CD8
CD4

Supplementary Figure 5. **Differentiation of 3PPE into TECs with the capacity to promote T cell production**

(A) control of transcriptional regulator FOXN1 of TECs was detected by flow cytometry under TEC-2 culture condition respectively at day 13 (FOXN1-PE-CF594). **(B)** control of mature TEC marker HLA-DRA (MHC class II molecule) and EpCAM were detected by qRT-PCR (Left panel) under six different TEC culture conditions and by flow cytometry (Right panel) under TEC-2 culture condition respectively at day 13(MHC-II-Alexa Fluor 488, EpCAM-PE). **(C)** control of cells for medullary TEC markers K5 and Aire were measured by flow cytometry under TEC-2 culture condition at day 13 (K5-Alexa Fluor 488, AIRE-Alexa Fluor 488). **(D)** The positive proportions of cells for medullary TEC markers AIRE and FOXN1 were measured by flow cytometry under TEC-2 culture condition at day 13. **(E)** control of CD205 cells and EpCAM cells were measured by flow cytometry under TEC-2 culture condition at day 13 (CD205-APC, EpCAM-PE). **(F)** hESCs (Day 0) was stained by immunofluorescence, Aire (Green) and DAPI was used to stain the nucleus (Blue). Scale bar = 200 μm. (**G**) The endoderm of the third pharyngeal pouch (Day 9) was stained by immunofluorescence, Aire (Red) and EpCAM (Green), and DAPI was used to stain the nucleus (Blue). Scale bar = 50 μm. **(H)** T cells gating strategy of CD34+ cells group and CD34+ cells group plus TEC group.

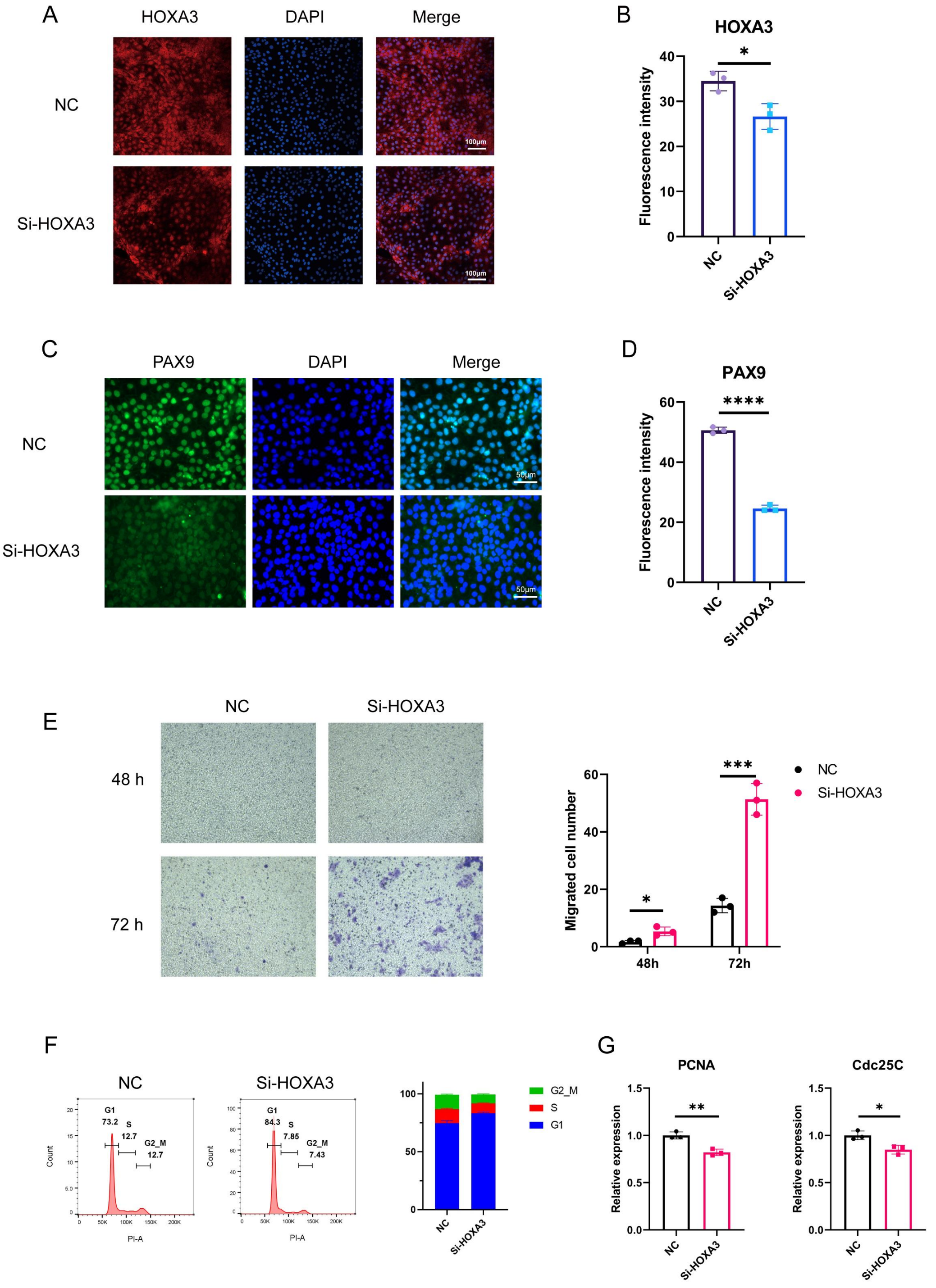
A
HOXA3
DAPI
Merge
NC
Si-HOXA3
100μm
B
HOXA3
Fluorescence intensity
*
NC
Si-HOXA3
C
PAX9
DAPI
Merge
NC
Si-HOXA3
50μm
D
PAX9
Fluorescence intensity
****
NC
Si-HOXA3
E
NC
Si-HOXA3
48 h
72 h
Migrated cell number
*
***
48h
72h
F
NC
Si-HOXA3
G1
73.2
S
12.7
G2_M
12.7
G1
84.3
S
7.85
G2_M
7.43
Count
PI-A
G2_M
S
G1
G
PCNA
Cdc25C
Relative expression
**
*

Supplemental Figure 6. HOXA3 regulated the differentiation, migration and proliferation of 3PPE

(A, B) Representative confocal images of double immunostaining for HOXA3 (Red) and EpCAM (Green) in cells treated with HOXA3 siRNA (A). Scale bar = 50 μm. The immunofluorescence of HOXA3 protein staining were quantification of (B). Statistical analysis by t test. **(C, D)** Representative confocal images of immunostainings of PAX9 (C) in cells treated with HOXA3 siRNA, and DAPI was used to stain the nucleus (blue). Scale bar = 50 μm. The immunofluorescence of PAX9 protein staining were quantification of (D). Statistical analysis by t test. **(E)** Representative images (Left panel) and quantitation (Right panel) of cell migration assays using transwells for the measurement of cell migration 72 hours after the treatment with HOXA3 siRNA. Statistical analysis by t test. **(F)** Cell cycle distributions were determined by flow cytometry with propidium iodide staining 48 hours after the treatment with HOXA3 siRNA. Statistical analysis by t test. **(G)** The expression changes of proliferation-related genes PCNA and cdc25c were determined by qRT-PCR in cells 48 hours after the treatment with HOXA3 siRNA (n=3). Statistical analysis by t test. Values are presented in mean ± SD. *$p < 0.05$, **$p < 0.01$, ***$p < 0.001$, ****$p < 0.0001$. n=3 independent biological repeats.

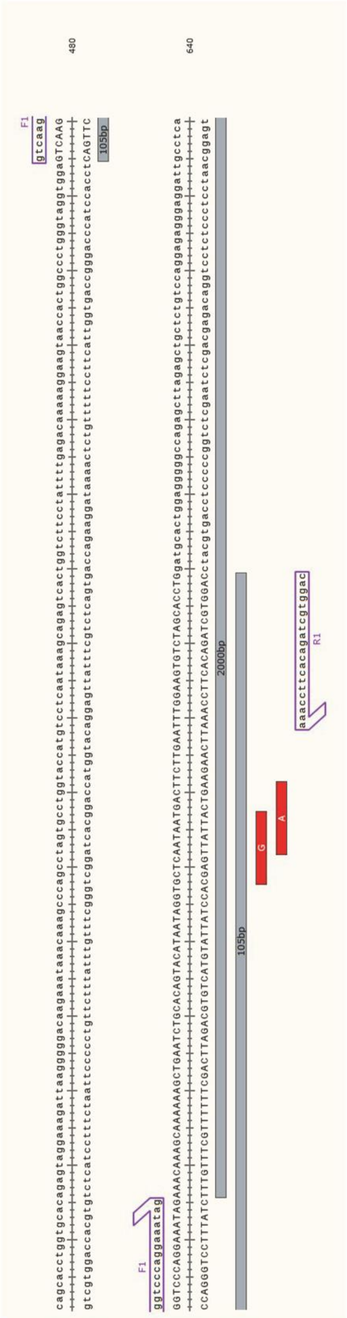
F1
gtcaag
ggtcccaggaaatag
R1
aaacttcacagatcgtggac
105bp
2000bp
G
A
480
640

Supplementary Figure 7. Binding site of transcription factor HOXA3 in EPHB2 promoter region

The red positions represented the binding sites of transcription factor HOXA3 in EPHB2 promoter.

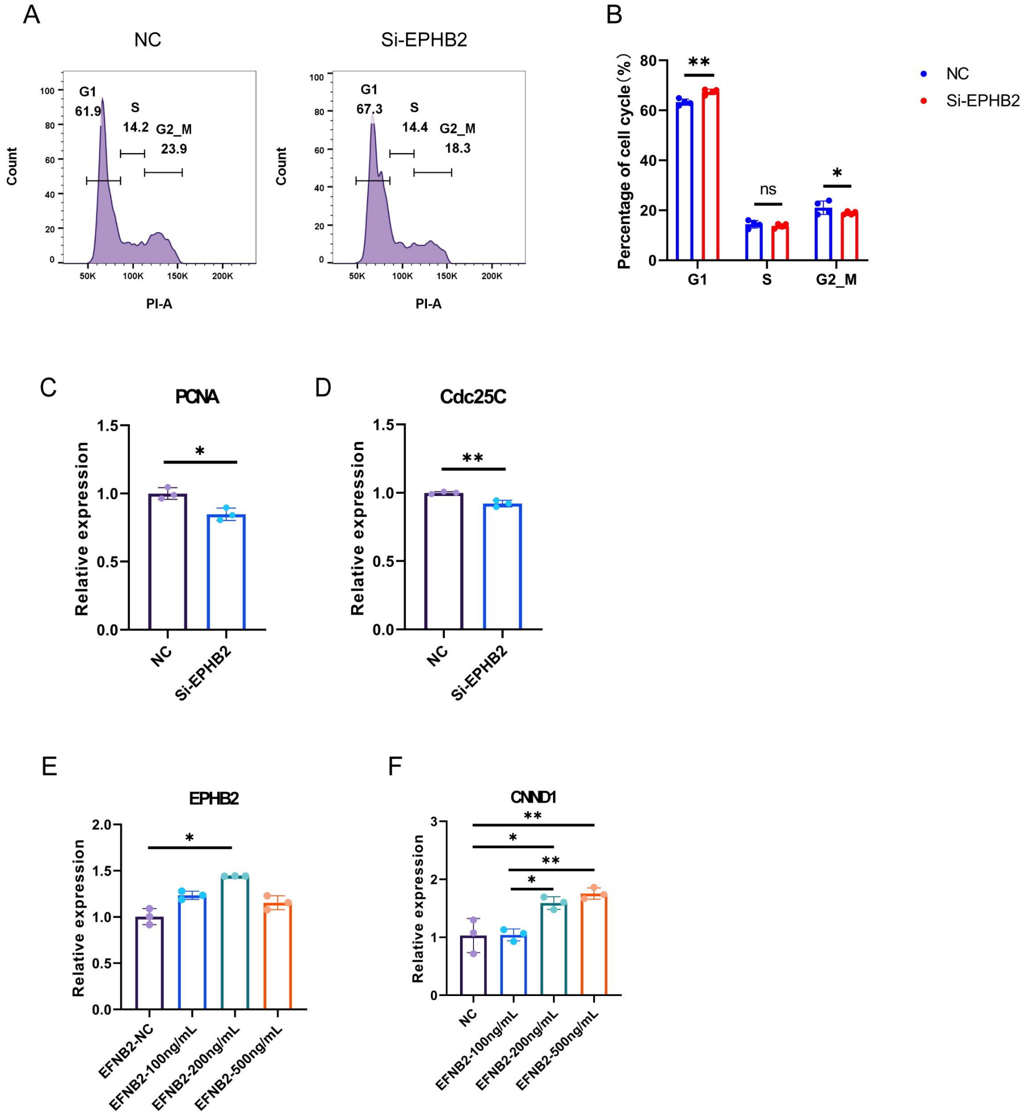
A
NC
Si-EPHB2
G1
61.9
S
14.2
G2_M
23.9
G1
67.3
S
14.4
G2_M
18.3
Count
PI-A
B
Percentage of cell cycle (%)
NC
Si-EPHB2
G1
S
G2_M
ns
C
PCNA
D
Cdc25C
Relative expression
NC
Si-EPHB2
E
EPHB2
F
CNND1
EFNB2-NC
EFNB2-100ng/mL
EFNB2-200ng/mL
EFNB2-500ng/mL

Supplementary Figure 8. Effects of EPHB2 on cell cycle of 3PPE and effects of EFNB2 on enhancing the expression of EPHB2 and CNND1

(A, B) Cell cycle distributions were determined by flow cytometry with propidium iodide staining 48 hours after the treatment with EPHB2 siRNA (n=4). Statistical analysis by t test. **(C, D)** The expression changes of proliferation-related genes PCNA (C) and Cdc25c (D) were determined by qRT-PCR in cells 48 hours after the treatment with EPHB2 siRNA (n=3). Statistical analysis by t test. **(E, F)** The expression changes of EPHB2 (E) and CNND1 (F) were assessed qRT-PCR after the treatment of cells with different concentrations of EFNB2 protein (n=3). Statistical analysis by ANOVA with Tukey's multiple comparison test. Values were presented in mean ± SD. *$p < 0.05$, **$p < 0.01$, ***$p < 0.001$, ****$p < 0.0001$.

Supplementary table 1. Different differentiation conditions for thymic epithelial cells (TEC) derived from 3PPE cells

**TEC-1**<br>DMEM-F12<br>0.5%B27 5ng/mL FGF10<br>1μM RA 50ng/mL WNT3A<br>50ng/mL FGF8 10ng/mL BMP4<br>10ng/mL Heparin Sodium<br>50μg/mL 2-Phospho-L-ascorbic acid trisodium salt	**TEC-2**<br>DMEM-F12<br>0.5%B27 5ng/mL FGF10<br>1μM RA 50ng/mL WNT3A<br>50ng/mL FGF8 10ng/mL BMP4<br>10ng/mL Heparin Sodium<br>50μg/mL 2-Phospho-L-ascorbic acid trisodium salt<br>10μM SB431542
**TEC-3**<br>DMEM-F12<br>0.5%B27 5ng/mL FGF10<br>1μM RA 50ng/mL WNT3A<br>50ng/mL FGF8 10ng/mL BMP4<br>10ng/mL Heparin Sodium<br>50μg/mL 2-Phospho-L-ascorbic acid trisodium salt<br>2.5μmol/L SANT1	**TEC-4**<br>DMEM-F12<br>0.5%B27 5ng/mL FGF10<br>1μM RA 50ng/mL WNT3A<br>50ng/mL FGF8 10ng/mL BMP4<br>10ng/mL Heparin Sodium<br>50μg/mL 2-Phospho-L-ascorbic acid trisodium salt<br>2.5μmol/L SANT1<br>10μM SB431542
**TEC-5**<br>DMEM-F12<br>0.5%B27 5ng/mL FGF10<br>1μM RA 50ng/mL FGF8<br>10ng/mL BMP4<br>10ng/mL Heparin Sodium<br>50μg/mL 2-Phospho-L-ascorbic acid trisodium salt	**TEC-6**<br>DMEM-F12<br>0.5%B27 5ng/mL FGF10<br>1μM RA 50ng/mL FGF8<br>10ng/mL BMP4<br>10ng/mL Heparin Sodium<br>50μg/mL 2-Phospho-L-ascorbic acid trisodium salt<br>2.5μmol/L SANT1<br>10μM SB431542

Supplementary table 2. Predicted putative binding sites of HOXA3 in EPHB2 promoter region

Matrix ID	Name	Score	Relative score	Sequence ID	Start	End	Strand	Predicted sequence
UN0558.1	UN0558.1.HOXA3	10.0628	0.942480027580693	seq1	192	201	-	AGTCATTATT
UN0558.1	UN0558.1.HOXA3	7.2731996	0.88892367397331831	seq1	983	992	-	CATCATTAAC
UN0558.1	UN0558.1.HOXA3	6.6335845	0.8770288252199241	seq1	1481	1490	-	TGCAATGAAT
UN0558.1	UN0558.1.HOXA3	6.502189	0.8745209682590441	seq1	983	992	+	GTTAATGATG
UN0558.1	UN0558.1.HOXA3	5.471121	0.8548416411476419	seq1	192	201	+	AATAATGACT
UN0558.1	UN0558.1.HOXA3	4.1482077	0.8295920622619802	seq1	1143	1152	+	ACCCATGGAC
UN0558.1	UN0558.1.HOXA3	3.5093596	0.8173987868345027	seq1	995	1004	+	GGTGATGAAG
UN0558.1	UN0558.1.HOXA3	3.4983974	0.8171895575758323	seq1	1481	1490	+	ATTCATTGCA
UN0558.1	UN0558.1.HOXA3	3.317623	0.8137392338150554	seq1	188	197	+	GCTCAATAAT
UN0558.1	UN0558.1.HOXA3	2.846049	0.8047386123960125	seq1	928	937	+	GGTGATTGCC

Supplementary table 3. Primers for qPCR and PCR used in this study

Gene	Forward Primer (5' to 3')	Reverse Primer (5' to 3')
GAPDH	GAAGATGGTGATGGGATTTC	GAAGGTGAAGGTCGGAGTC
OCT4	AGGCAACCTGGAGAATTTGTTC	CACACTCGGACCACATCCTTC
NANOG	CAGGACAGCCCTGATTCTTCC	TTTGCGACACTATTCTCTGCAGA
SOX2	GCCGAGTGGAAACTTTTGTCG	GGCAGCGTGTACTTATCCTTCT
BRACHYURY	TGTCCCAGGTGGCTTACAGATGAA	GGTGTGCCAAAGTTGCCAATACAC
CXCR4	AACCAGCGGTTACCATGGAG	CACGGAAACAGGGTTCCTTCA
SOX17	GGCGCAGCAGAATCCAGA	CCACGACTTGCCCAGCAT
FOXA2	GGGAGCGGTGAAGATGGA	TCATGTTGCTCACGGAGGAGTA
GATA4	CGACTTCTCAGAAGGCAGAGAGTG	CTTCATGTAGAGGCCGCAGGCATT
TBX1	TAGCGAGAAATATGCCGAGGA	CGTGATCCGATGGTTCTGGT
SIX1	CTGCCGTCGTTTGGCTTTAC	GCTCTCGTTCTTGTGCAGGT
PAX8	ATCCGGCCTGGAGTGATAGG	TGGCGTTTGTAGTCCCCAATC
PBX1	GACAACTCAGTGGAGCATTCA	CTCTCGCAGGAGATTCATCAC
FGF4	CTCGCCCTTCTTCACCGATG	GTAGGACTCGTAGGCGTTGTA
CER1	GGATGGCCGCCAGAATCAG	TGGCACTGCGACAAACAGAT
EYA1	GTCACAGTCTCAGAGACACCTGG	GGGATAAGACGGATAGTCCTGC
PAX9	GGCGTGTGCGACAAGTACA	GGGCCAAGTTGCCGATCTT
HOXA3	ATGCAAAAAGCGACCTACTACG	TGCAAAAAGCGACCTACTACG
FOXN1	GAGAGTGGTGCTGGGATGTT	ATGGGTTTTGGGAAGAGAGG
MHC-II	AAGCAATGCAGCAGAACGC	GTAAAGCCATTAAAGCAGAAG
KRT5	ATTCTGCTTCAAATCAGCCTTCA	AACTTTGGGTTCTCGTGTCAGC
AIRE	GCGGGAGAGGAGGTAAGAG	AGGACCCACACACAGTAGGG
CTHRC1	CAATGGCATTCCGGGTACAC	GTACACTCCGCAATTTTCCCAA
INSL3	ACCCCAGAGATGCGTGAGAA	CTCCAGCCACTGTAGCAACTC
PCNA	CAAGTAATGTCGATAAAGAGGAGG	GTGTCACCGTTGAAGAGAGTGG

MCM2	AGATGGCATGGAAAGGGAC	GTCCTCATCATCCAGAGCC
CDC45L	TGGATGCTGTCCAAGGACCTGA	CAGGACACCAACATCAGTCACG
Cdc25C	AGAAGCCCATCGTCCCTTTGGA	GCAGGATACTGGTTCAGAGACC
c-Myc	AGCTCATTTCTGAAGAGGACTTGT	TTGAGGCAGTTTACATTATGGCTA
Wnt9A	AGCAGCAAGTTCGTCAAGGAA	CCTTCACACCCACGAGGTTG
WNT1	CTCTTCGGCAAGATCGTCAACC	CGATGGAACCTTCTGAGCAGGA
FZD3	GTTCATGGGGCATATAGGTGG	GCTGCTGTCTGTTGGTCATAA
FZD8	GCTCTACAACCGCGTCAAGACA	AAGGTGGACACGAAGCAGAGCA
FZD5	CCGTTCGTGTGCAAGTGTC	GAAGCGTTCCATGTCGATGAG
FRAT1	ACAGAGTAGCCAGGTTCTG	AACAAGCCATTGACGAAGC
GSK3β	GGCAGCATGAAAGTTAGCAGA	GGCGACCAGTTCTCCTGAATC
TCF12	CCAGTAGTTATGGCAACCTTCAT	GACTCGTGTTTATGTCTGTTGGT
CCND1	CAATGACCCCGCACGATTTC	CATGGAGGGCGGATTGGAA
EPHB2	AGAAACGCTAATGGACTCCACT	GTGCGGATCGTGTTCATGTT
NC	UUCUCCGAACGUGUCACGUDTDT	ACGUGACACGUUCGGAGAADTDT
Si-HOXA3	CGGUGGCUAUCUGAACUCUAUTT	AUAGAGUUCAGAUAGCCACCGTT
Si-EPHB2	GCACACCUGUGAUGAUCAUDTDT	AUGAUCAUCACAGGUGUGCDTDT
EPHB2-promoter	GTCAAGGGTCCCAGGAAATAG	CAGGTGCTAGACACTTCCAAA

Supplementary table 4. Antibodies used in this study

Antigen	Source	Cat#
FoxA2	CST	8186S
SIX1	CST	12891S
PAX9	CST	12847S
EpCAM	CST	2929S
FOXN1	Santa Cruz	sc-271256
Keratin 5	CST	25807S
Keratin 8	CST	4546s
MHC Class II	abcam	ab55152
AIRE	abcam	ab65040
HOXA3	bioss	bs-11292R
BMP4	CST	4680S
GAPDH	CST	5174S
Frizzled 8	bioss	bs-13219R
GSK3B	CST	12456T
Phospho-GSK3B	CST	5558T
Beta catenin	bioss	bs-23663R
Cyclin D1	CST	55506T
c-Myc	bioss	bs-24507R
EphB2	CST	83029T
HOXA3	Santa Cruz	sc-374237 X
PE Anti-Human OCT4 (OCT3) Antibody	STEM CELL	60093PE
APC/Cyanine7 anti-human CD184 (CXCR4)	Biolegend	306528
PE Mouse anti-Human Sox17 Clone P7-969 (RUO)	Biolegend	561591
Alexa Fluor 488 anti-human HLA-DR	Biolegend	307619
PE anti-human CD326 (Ep-CAM)	Biolegend	324206

APC anti-human CD205	Biolegend	342207
APC/Cy7 anti-human CD45	Biolegend	304014
Brilliant Violet 510™ anti-human CD3	Biolegend	317332
Alexa Fluor® 700 anti-human CD8a	Biolegend	300920
PE anti-human CD4	Biolegend	317410
Brilliant Violet 421™ anti-human TCR α/β	Biolegend	306721
Anti-mouse IgG Fab2 Alexa Fluor (R) 488	CST	4408S
Anti-rabbit IgG Fab2 Alexa Fluor (R) 594	CST	8889S
Anti-mouse IgG Fab2 Alexa Fluor (R) 594	CST	8890S
Anti-rabbit IgG Fab2 Alexa Fluor (R) 488	CST	4412S
HRP-labeled Goat Anti-Rabbit IgG(H+L)	Beyotime	A0208
HRP-labeled Goat Anti-Mouse IgG(H+L)	Beyotime	A0216
